# Drought resistance and post-rewatering recovery of *Dendropanax trifidus* under elevated CO_2_ and temperature are mediated by photoprotective regulation and carbon reallocation

**DOI:** 10.1186/s12915-026-02707-3

**Published:** 2026-08-14

**Authors:** Jong Chan Yeo, Yeong Geun Song, Mark Bryan Carayugan, Hyun Ju Lee, Woo Bin Youn, Kyeong Cheol Lee, Ji Young An, Byung Bae Park

**Affiliations:** 1 Department of Environment and Forest Resources, College of Agriculture and Life Sciences, Chungnam National University, Daejeon, 34134 Republic of Korea; 2 Department of Forestry, College of Agriculture and Life Sciences, Jeonbuk National University, Jeonju, 54896 Republic of Korea; 3 Forest Environment & GeoSpatial Technology Research Institute, Sejong, 30084 Republic of Korea; 4 Department of Crops and Forestry, Korea National University of Agriculture and Fisheries, Jeonju, 54874 Republic of Korea; 5 Division of Environmental and Forest Science, College of Agriculture and Life Sciences, Gyeongsang National University, Jinju, 52828 Republic of Korea

**Keywords:** *Dendropanax trifidus*, Climate change scenarios, Drought resistance, Post-drought recovery, Photosynthetic electron transport, Carbon allocation

## Abstract

**Background:**

Drought is expected to become more frequent and severe under climate change, affecting forest structure and carbon sequestration. Tree responses involve multiple physiological and morphological adjustments, but the integrated mechanisms governing drought resistance and post-drought recovery under future climate scenarios remain insufficiently understood. We investigated drought resistance and recovery responses of *Dendropanax trifidus* seedlings, a warm-temperate species, under three shared socio-economic pathway (SSP) scenarios reflecting elevated CO_2_ and temperature by the late twenty-first century.

**Results:**

During drought exposure, increased absorption flux (ABS/RC) and dissipated energy flux (DI_*o*_/RC) per reaction center indicated enhanced photoprotective energy dissipation. Concurrently, decreases in net photosynthetic rate (*P*_*n*_) and stomatal conductance (*g*_*s*_) showed restricted gas exchange and carbon assimilation, while biomass allocation shifted toward roots to prioritize water acquisition. After rewatering, increased maximum quantum yield of photosystem II (*F*_*v*_/*F*_*m*_) and decreased ABS/RC and DI_*o*_/RC indicated alleviation of the drought-induced photoprotective state. These changes were accompanied by increases in *P*_*n*_, *g*_*s*_, and aboveground biomass reallocation. Notably, *P*_*n*_ failed to return to pre-drought levels under SSP1 but exceeded them under both SSP3 and SSP5, suggesting that scenario-specific environmental conditions influenced photosynthetic recovery.

**Conclusions:**

These findings suggest that *D. trifidus* drought resistance depends primarily on photosystem protection, reduced water loss, and root-centered resource allocation, rather than maintaining photosynthetic carbon gain. Post-rewatering recovery involves alleviating photoprotective regulation, restoring gas exchange and carbon assimilation, and shifting biomass allocation toward aboveground organs.

**Supplementary Information:**

The online version contains supplementary material available at https://doi.org/10.1186/s12915-026-02707-3.

## Background

Compound drought and heat extremes have become increasingly prevalent in recent decades as global CO_2_ emissions and the impacts of climate change have intensified [1]. Shifting climatic baselines have led to increasingly frequent “hotter drought” conditions, which are now recognized globally as a major driver of widespread forest mortality across diverse biomes [2, 3]. Up to 71% of forest net primary productivity may be at risk of depletion due to these compound extremes, leading to extensive tree mortality, canopy dieback, and declines in forest carbon uptake and sequestration capacity [4, 5]. Abiotic stresses induced by climatic anomalies constrain plant functional processes, thereby altering species demographics and community structure by (1) reducing suitable ecological niches for plant species with narrow tolerance limits and (2) opening new habitats for species with broader ecological amplitudes [6, 7]. Many climate models predict that the frequency and intensity of drought will further increase as atmospheric CO_2_ concentration and atmospheric temperature rise. In response, plants may regulate their hydraulic processes and net photosynthetic rate, potentially constraining carbon exchange between vegetation and the atmosphere [8, 9]. Therefore, a clear understanding of seedling responses under deeply intertwined climatic stressors is essential for elucidating (1) the links between plant growth and function and concurrent climatic anomalies, and (2) plant tolerance and adaptation mechanisms under continuously changing CO_2_ concentrations, atmospheric temperatures, and water regimes.

Plant responses to drought and heat are regulated through interacting hydraulic, photosynthetic, and photoprotective processes [10, 11]. From the perspective of contemporary plant hydraulics, woody plant mortality under such extreme conditions is largely driven by the complex interdependence between hydraulic failure and carbon starvation. This process is further exacerbated by the combined effects of rising atmospheric CO_2_, elevated temperature, severe soil drought, and high vapor pressure deficit (VPD), as well as their complex interactions [12]. However, response pathways to multiple stressors are often nonlinear and highly species-specific [10]. Some plant species rely on hydraulic regulation by initiating stomatal closure, thereby minimizing transpirational water loss, maintaining higher leaf and xylem water potentials, and preventing xylem embolism-induced hydraulic dysfunction that leads to drought-induced tree mortality [13]. Elevated atmospheric CO_2_ is expected to partially alleviate drought stress by downregulating stomatal conductance and improving intrinsic water use efficiency [14], although the actual soil water-saving effect may be offset by compensatory structural responses, such as increases in leaf area [15]. In contrast, another response observed in plants under adequate soil moisture conditions is enhanced or prolonged transpiration at the onset of compound extremes [16]. Transpirational water loss can serve as a potential cooling mechanism that regulates plant temperature below thermal physiological limits, protects the leaf photosynthetic apparatus—particularly the components involved in electron transport—and maintains thylakoid membrane fluidity [16, 17]. Plant species primarily adopt a photoprotective strategy by modifying chlorophyll fluorescence responses to reduce inefficient photochemical reactions [11, 18, 19]. Numerous previous studies have reported a strategic reduction in the maximum quantum yield of photosystem II (*F*_*v*_/*F*_*m*_), accompanied by sharp increases in absorption flux per reaction center (ABS/RC), dissipated energy flux per reaction center (DI_*o*_/RC), electron transport flux from the primary plastoquinone acceptor to the secondary quinone acceptor (ET_*o*_/RC), and electron flux to Photosystem I acceptors (RE_*o*_/RC) under combined drought and heat stress [11, 20, 21]. These changes indicate a temporary downregulation of reaction centers and non-photochemical dissipation of excess excitation energy as heat. Such restrictions in the photosynthetic apparatus may temporarily minimize carbon assimilation, leading to reduced carbohydrate production, biomass accumulation, and plant growth [9]. Given the diverse and complex strategies that plants use to cope with CO_2_-induced compound extremes, clarifying species-specific responses and mechanisms of drought resistance and recovery is crucial for understanding plant survival under climate change.

Monitoring biomass investment pathways, along with carbon uptake and storage, is also critical, as these processes contribute to stress resistance and post-stress recovery under concurrent drought and warming [22]. In particular, changes in biomass allocation patterns reflect the functional priorities of plants facing environmental stress [23]. Many previous studies have shown that biomass is allocated in a greater proportion to belowground growth under drought stress, likely to promote deep root production and enhance water uptake [23–25]. Once drought stress is relieved, many plants redirect biomass toward aboveground development, promoting stem elongation and radial growth [26, 27]. However, these post-stress recovery trajectories may be altered under the increasing atmospheric CO_2_ enrichment observed in recent decades. Under non-limiting water conditions, elevated CO_2_ can facilitate rapid stomatal reopening, leading to enhanced carbon uptake, carbon fixation, and productivity [28]. Increased photosynthetic gain enables recovering plants to produce additional assimilates, which can be stored in stems—particularly in the parenchyma cells of the sapwood—as non-structural carbohydrates (NSCs) [29, 30]. These NSCs may support metabolic recovery and the repair of the hydraulic system after severe drought [31]. Most importantly, these reserves can function as osmotic regulators or carbon sources, providing plants with an additional survival strategy during subsequent drought events [31, 32]. Nevertheless, how plants coordinate these recovery strategies under the simultaneous pressures of elevated temperature and atmospheric CO_2_ remains insufficiently understood, highlighting the need for more detailed investigations of biomass allocation and carbon distribution patterns.

*Dendropanax trifidus* (Thunb.) Makino ex H. Hara is a warm-temperate tree species native to the Korean Peninsula. Its distribution is projected to expand under climate change, suggesting that it may tolerate relatively warm and dry conditions [33, 34]. As a native species with high ecological and silvicultural value, *D. trifidus* is increasingly used for urban greening and plantation establishment. Despite this importance, the processes underlying its capacity to maintain function under concurrent climate-change stressors remain poorly understood. Recent advances emphasize that understanding drought adaptation requires moving beyond isolated traits toward an integrated assessment of multiple functional processes governing both dehydration tolerance and dehydration avoidance [35]. In particular, little is known about how *D. trifidus* coordinates photoprotective regulation, gas exchange, biomass allocation, and carbon storage during drought exposure and after rewatering.

This study examined the changes in growth performance, physiological states, morpho-anatomical traits, and biochemical status of *D. trifidus* seedlings during the pre-drought, drought exposure, and post-rewatering recovery stages. Investigations were conducted under three shared socioeconomic pathway (SSP) scenarios proposed by the Intergovernmental Panel on Climate Change (IPCC) for the late twenty-first century (2080–2100): SSP1-2.6, SSP3-7.0, and SSP5-8.5 [36]. Through this approach, we sought to identify the drought resistance and recovery strategies of this warm-temperate tree species under the combined effects of elevated atmospheric CO_2_, increased temperature, and water stress. Given that the Korean Peninsula is warming faster than the global average [37], this region serves as an important proxy for observing the future impacts of accelerated climate change. Therefore, the findings of this study provide insights into the adaptive strategies of warm-temperate generalist species under extreme climate change and offer practical baseline information for developing effective tree management and afforestation practices in the Republic of Korea.

## Results

### Changes in growth performance according to watering treatment stages and SSP Scenarios

**Fig. 1:**
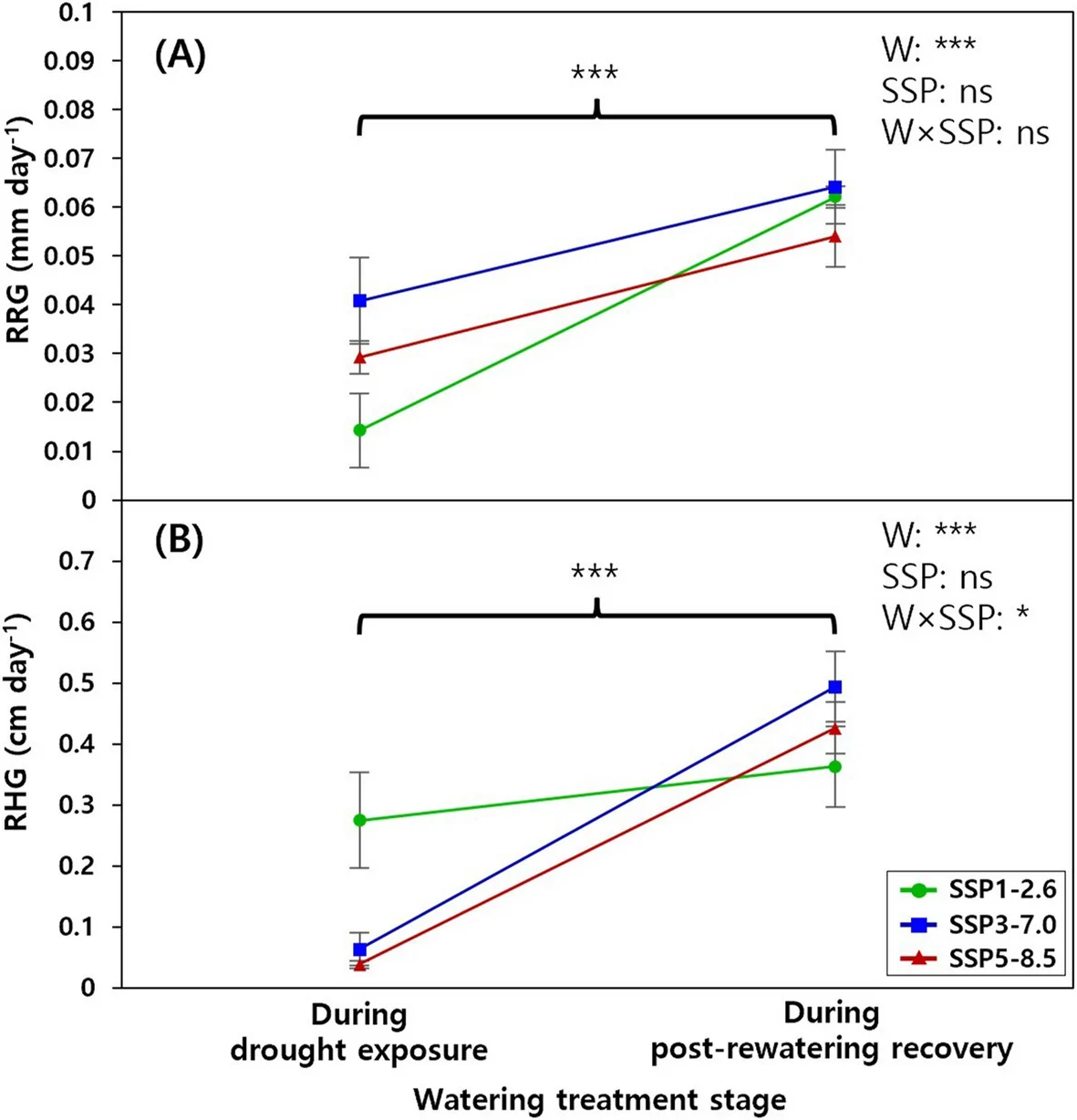
RRG and RHG of *Dendropanax trifidus* under different watering treatment and SSP scenarios. **A** Relative root collar diameter growth (RCD) rate (mm day^−1^) and **B** relative height growth rate (cm day^−1^) of *Dendropanax trifidus* during the drought exposure and post-rewatering recovery stages under different SSP scenarios. Vertical bars indicate standard error of the mean (*n* = 3). Asterisks indicate statistical significance (*p < 0.05, **p < 0.01, ***p < 0.001), and “ns” represents non-significance based on two-way repeated measures ANOVA followed by Bonferroni multiple comparisons test

For relative root collar diameter growth rate (RRG), the interaction between watering treatment stage and SSP scenario was not significant (*p* = 0.146). However, RRG was significantly different during the post-rewatering recovery stage than during the drought exposure stage across all scenarios (*p* = 0.001; Fig. 1A), with recovery values reaching up to 2.1 times those observed during the drought exposure stage. For relative height growth rate (RHG), a significant interaction was detected between watering treatment stage and SSP scenario (*p* = 0.003; Fig. 1B). RHG was generally higher during the post-rewatering recovery stage than during the drought exposure stage but the magnitude of this difference varied among SSP scenarios. Under SSP3 and SSP5, RHG increased markedly after rewatering. Under SSP1, RHG remained comparable between the drought exposure and post-rewatering recovery stages.

**Fig. 2:**
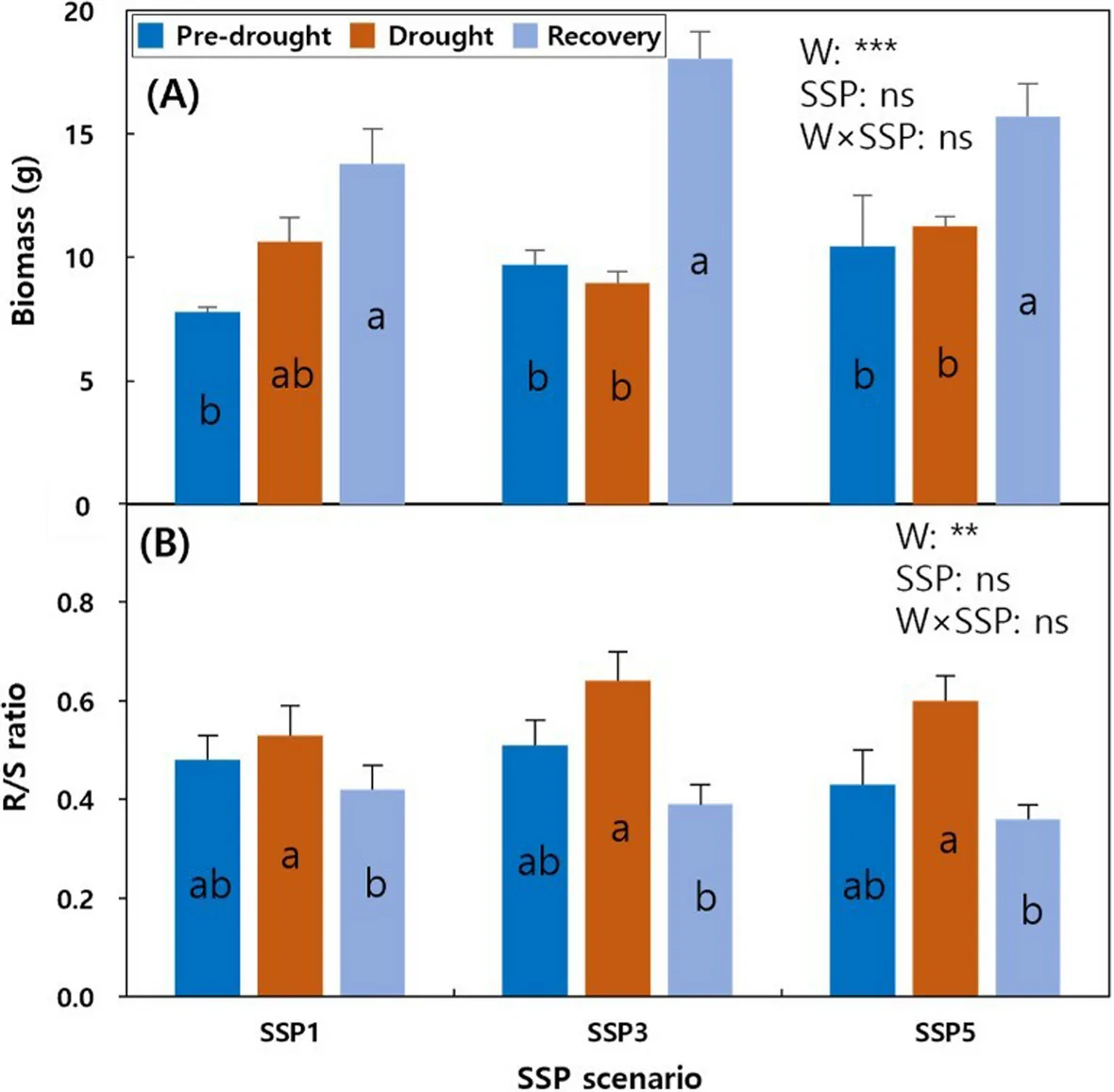
Total biomass and R/S ratio of *Dendropanax trifidus* under different watering treatment and SSP scenarios. The figures show **A** total biomass (g) and **B** root-to-shoot ratio of *Dendropanax trifidus* across watering treatment stages and SSP scenarios. The three watering treatment stages were the pre-drought stage (pre-drought in the figures), drought exposure stage (drought in the figures), and post-rewatering recovery stage (recovery in the figures). Vertical bars indicate standard error of the mean (*n* = 3). Lowercase letters indicate significant differences among watering treatment stages within the same SSP scenario. Asterisks indicate statistical significance (*p < 0.05, **p < 0.01, ***p < 0.001), and “ns” represents non-significance based on standard two-way ANOVA followed by Bonferroni multiple comparisons test

For total biomass accumulation in *Dendropanax trifidus*, no significant interaction was detected between watering treatment stage and SSP scenario (*p* = 0.119), and the accumulation rate differed significantly only among watering treatment stages (*p* < 0.001; Fig. 2A). During the drought exposure stage, seedlings showed significantly slow biomass accumulation. After rewatering, total biomass increased by approximately 3.19–9.09 g across the SSP scenarios.

For the root-to-shoot ratio, no significant interaction was detected between watering treatment stage and SSP scenario. The root-to-shoot ratio differed significantly among watering treatment stages (*p* = 0.002; Fig. 2B). The ratio reached its highest values during the drought exposure, increasing by 10.4–39.5% relative to the preceding stage. During the post-rewatering recovery, the ratio decreased by 20.8–40.0%.

### Changes in physiological states according to watering treatment stages and SSP Scenarios

**Fig. 3:**
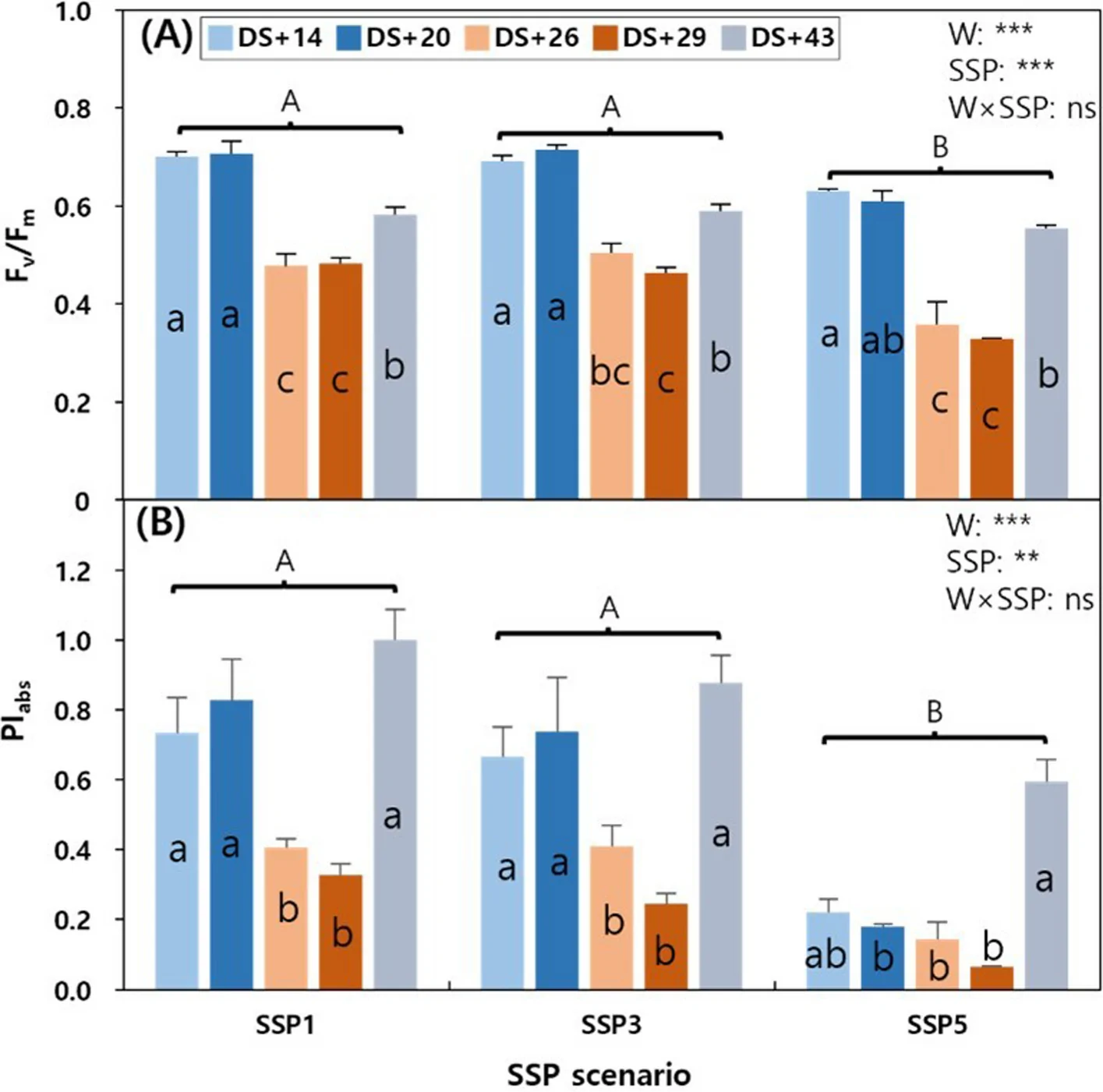
*F*_*v*_/*F*_*m*_ and PI_abs_ under five measurement stages and SSP scenarios. The figures show **A** maximum quantum efficiency of photosystem II (*F*_*v*_/*F*_*m*_) and **B** performance index on an absorption basis (PI_abs_) of *Dendropanax trifidus* under measurement stages and SSP scenarios. The five measurement stages include the following: early pre-drought stage (DS + 14), late pre-drought stage (DS + 20), mid-drought stage (DS + 26), peak-drought stage (DS + 29), and post-rewatering recovery stage (DS + 43). Vertical bars indicate the standard error of the mean (*n* = 3). Uppercase letters indicate significant differences among the SSP scenarios. Lowercase letters indicate significant differences among measurement stages within the same SSP scenario. Asterisks indicate statistical significance (*p < 0.05, **p < 0.01, ***p < 0.001), and “ns” indicates non-significance based on a two-way repeated measures ANOVA followed by the Bonferroni multiple comparisons test

For the maximum quantum yield of photosystem II (*F*_*v*_/*F*_*m*_) and the performance index on an absorption basis (PI_abs_), no significant interaction was detected between measurement stage and SSP scenario (*F*_*v*_/*F*_*m*_: *p* = 0.175; Fig. 3A, PI_abs:_ *p* = 0.062; Fig. 3B). However, both parameters differed significantly among measurement stages (*F*_*v*_/*F*_*m*_: *p* < 0.001; PI_abs_: *p* = 0.002) and SSP scenarios (*F*_*v*_/*F*_*m*_: *p* < 0.001; PI_abs_: *p* = 0.002). As drought exposure progressed, *F*_*v*_/*F*_*m*_ and PI_abs_ generally declined, reaching their minimum values at the peak-drought stage (Day of study 29; DS + 29). Across all SSP scenarios, *F*_*v*_/*F*_*m*_ decreased on average by 37.4% from the late pre-drought stage (DS + 20) to the peak-drought stage (DS + 29). Over the same period, PI_abs_ decreased on average by 63.8%. During the post-rewatering recovery stage (DS + 43), *F*_*v*_/*F*_*m*_ gradually increased across all SSP scenarios; however, it failed to recover to its initial level by the final measurement stage. Across the experimental period, *F*_*v*_/*F*_*m*_ and PI_abs_ were consistently lower under SSP5 than under the other SSP scenarios.

**Fig. 4:**
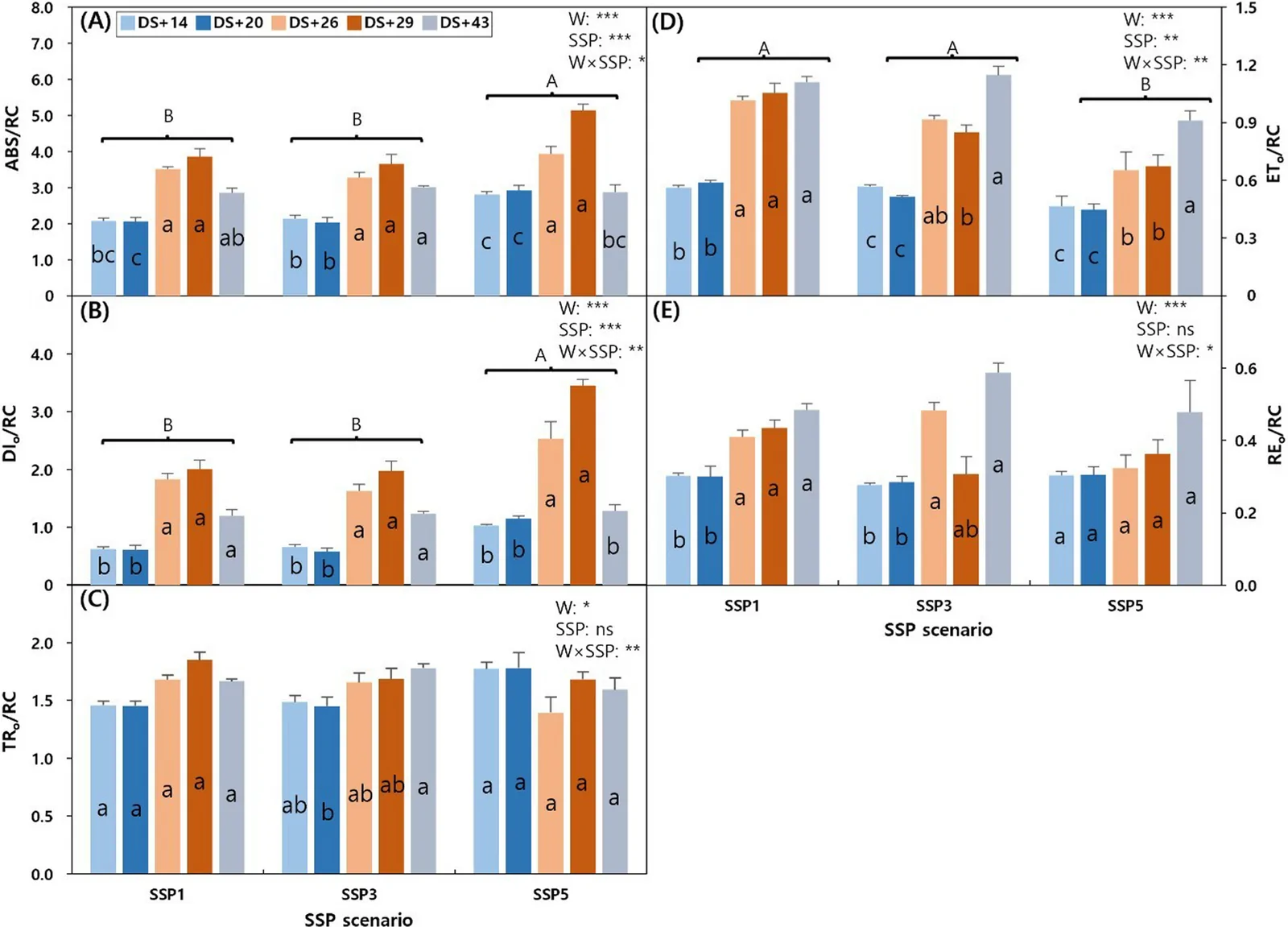
Specific energy flux parameters under five measurement stages and SSP scenarios. The figures show **A** ABS/RC, **B** DI_*o*_/RC, **C** TR_*o*_/RC, **D** ET_*o*_/RC, and **E** RE_*o*_/RC of *Dendropanax trifidus* under measurement stages and SSP scenarios. Refer to Table 1 for the definition of each specific energy flux parameter. The five measurement stages include the following: early pre-drought stage (DS + 14), late pre-drought stage (DS + 20), mid-drought stage (DS + 26), peak-drought stage (DS + 29), and post-rewatering recovery stage (DS + 43). Vertical bars indicate the standard error of the mean (*n* = 3). Uppercase letters indicate significant differences among SSP scenarios, whereas lowercase letters indicate significant differences among measurement stages within the same SSP scenario. Asterisks indicate statistical significance (*p < 0.05, **p < 0.01, ***p < 0.001), and “ns” represents non-significance based on two-way repeated measures ANOVA followed by Bonferroni multiple comparisons test

Significant interaction effects between measurement stage and SSP scenario were detected for all specific energy fluxes per reaction center including ABS/RC (*p* = 0.024), DI_*o*_/RC (*p* = 0.007), TR_*o*_/RC (*p* = 0.002), ET_*o*_/RC (*p* = 0.001), RE_*o*_/RC (*p* = 0.03; Fig. 4A–E). Absorption flux per reaction center (ABS/RC; Fig. 4A) and dissipated energy flux per reaction center (DI_*o*_/RC; Fig. 4B) increased during drought exposure, with the largest increases observed under SSP5 scenario. At the peak-drought stage (DS + 29), ABS/RC and DI_*o*_/RC under SSP5 were 1.3- and 1.7-fold higher, respectively, than at the late pre-drought stage (DS + 20). During the post-rewatering recovery stage (DS + 43), ABS/RC and DI_*o*_/RC under SSP5 decreased by 44.0% and 62.9% compared with DS + 29. These decreases were the largest among the SSP scenarios.

Trapped energy flux per reaction center (TR_*o*_/RC; Fig. 4C) remained relatively stable across the experimental period under most scenarios except SSP3. However, under SSP3, TR_*o*_/RC at DS + 29 and DS + 43 was 1.16 and 1.23 times higher, respectively, than at the late pre-drought stage (DS + 20). Electron transport flux per reaction center (ET_*o*_/RC; Fig. 4D) and electron flux reducing end electron acceptors at the Photosystem I acceptor side per reaction center (RE_*o*_/RC; Fig. 4E) generally increased from the early pre-drought stage (DS + 14) to the post-rewatering recovery stage (DS + 43). The increase in RE_*o*_/RC was significant under SSP1 and SSP3, but not under SSP5. Under SSP5, RE_*o*_/RC remained relatively stable, ranging from 0.39 to 0.49 across measurement stages.

**Fig. 5:**
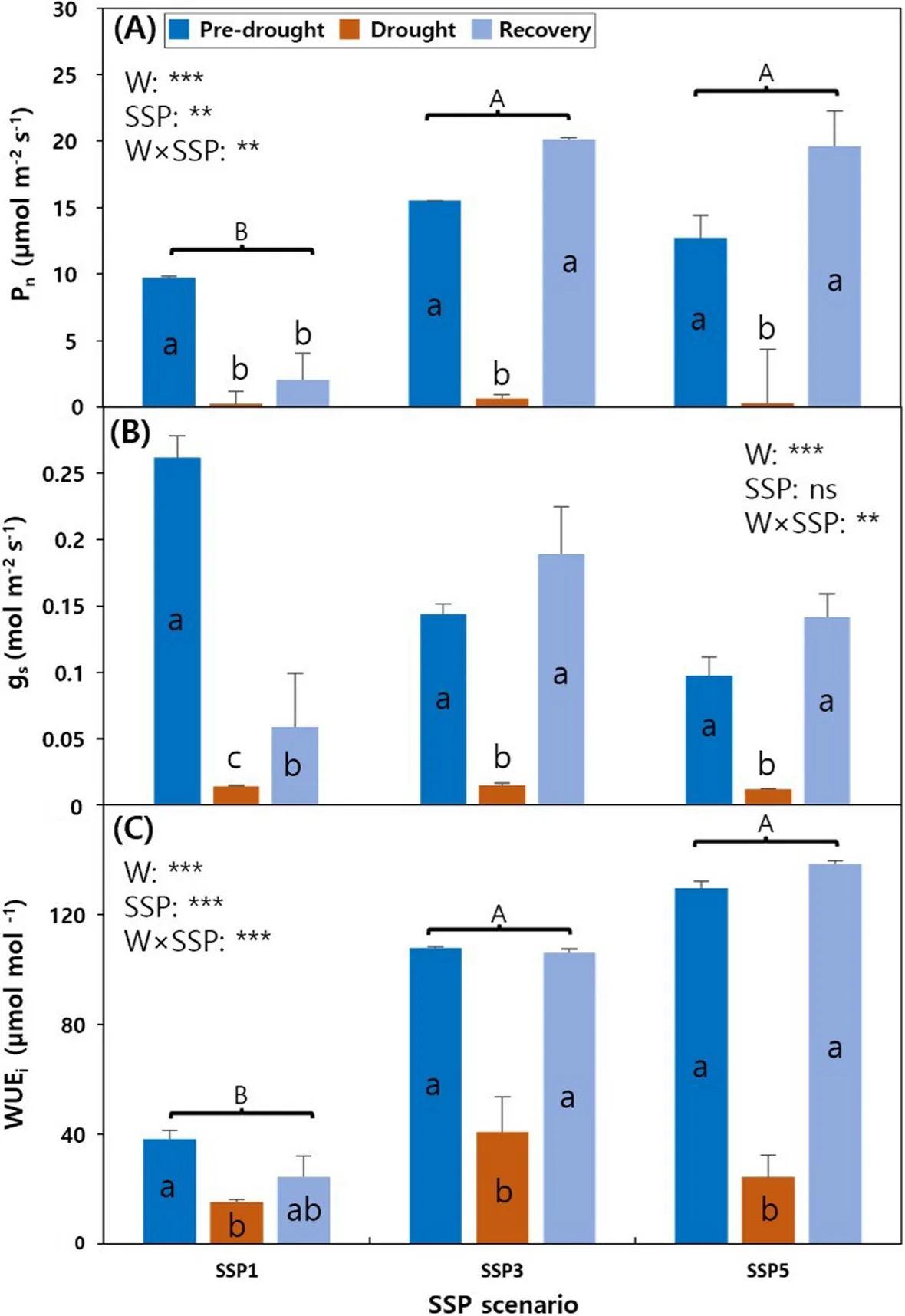
Leaf gas exchange parameters across watering treatment stages and SSP scenarios. The figures show **A** net photosynthetic rate (*Pₙ*), **B** stomatal conductance (*gₛ*), and **C** intrinsic water use efficiency (WUE_*i*_) of *Dendropanax trifidus* across watering treatment stages and SSP scenarios. The three watering treatment stages were the pre-drought stage (pre-drought in the figures), drought exposure stage (drought in the figures), and post-rewatering recovery stage (recovery in the figures). Vertical bars indicate the standard error of the mean (*n* = 3). Uppercase letters indicate significant differences among the SSP scenarios. Lowercase letters indicate significant differences among watering treatment stages within the same SSP scenario. Asterisks indicate statistical significance (*p < 0.05, **p < 0.01, ***p < 0.001), and “ns” represents non-significance based on two-way repeated measures ANOVA followed by Bonferroni multiple comparisons test

Leaf gas exchange parameters showed significant interactions between watering treatment stage and SSP scenario (*P*_*n*_: *p* = 0.003; *g*_*s*_: *p* = 0.008; WUE_*i*_: *p* = 0.043; Fig. 5A–C). During the drought exposure stage, net photosynthetic rate (*P*_*n*_), stomatal conductance (*g*_*s*_), and intrinsic water use efficiency (WUE_*i*_) decreased across all SSP scenarios compared with the pre-drought stage. *P*_*n*_ declined to less than 3.7% of the pre-drought stage level across all scenarios (Fig. 5A). Similarly, g_s_ decreased to less than 12.0% of the initial values (Fig. 5B), while WUE_*i*_ concurrently declined to 18.8%–40.1% of the pre-drought stage level (Fig. 5C). During the post-rewatering recovery stage, the effects of rewatering on these parameters differed depending on the SSP scenario. Under both SSP3 and SSP5 scenarios, all three parameters (*P*_*n*_, *g*_*s*_, and WUE_*i*_) increased significantly compared with the drought exposure stage. Specifically, *P*_*n*_ reached 129.4% and 163.3% of the pre-drought stage levels under SSP3 and SSP5, respectively. In addition, *g*_*s*_ reached 131.3% and 143.9% of the pre-drought stage levels under SSP3 and SSP5, respectively. WUE_*i*_ corresponded to 99.1% and 106.7% of the pre-drought stage levels, respectively. Under SSP1 scenario, *P*_*n*_ and *g*_*s*_ failed to recover to pre-drought stage levels during the post-rewatering recovery stage. *P*_*n*_ reached only 57.9% of the pre-drought stage level, and *g*_*s*_ remained at 22.5% of the pre-drought stage value.

**Fig. 6:**
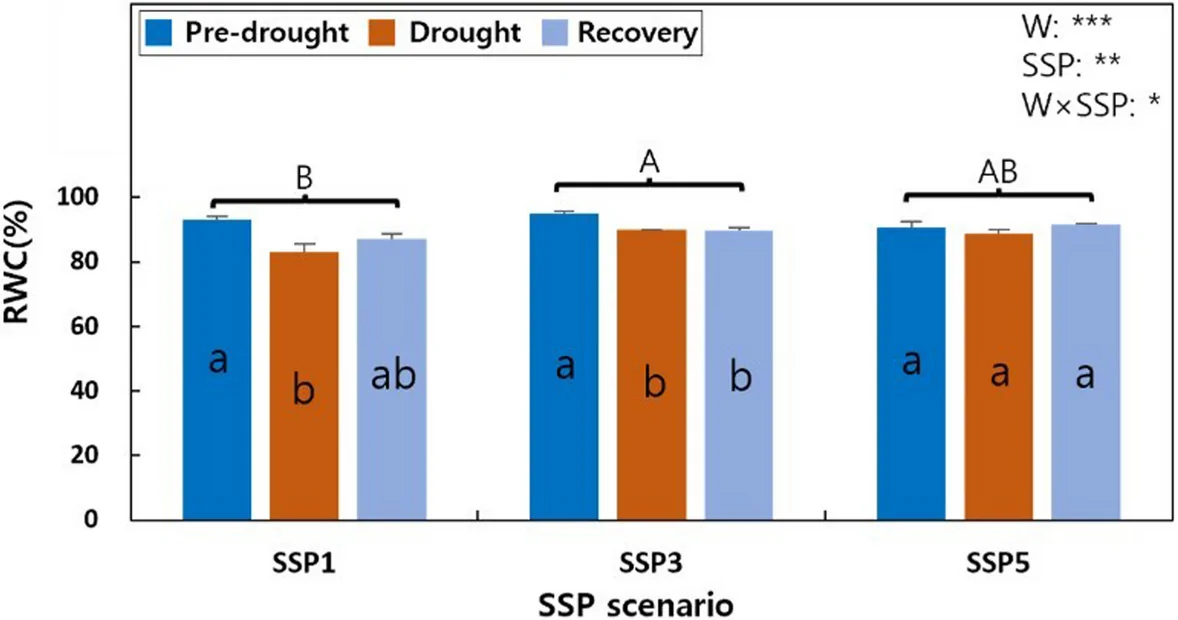
Leaf relative water content (RWC) across watering treatment stages and SSP scenarios. The three watering treatment stages were the pre-drought stage (pre-drought in the figure), drought exposure stage (drought in the figure), and post-rewatering recovery stage (recovery in the figure). Vertical bars indicate the standard error of the mean (*n* = 3). Uppercase letters indicate significant differences among the SSP scenarios. Lowercase letters indicate significant differences among watering treatment stages within the same SSP scenario. Asterisks indicate statistical significance (*p < 0.05, **p < 0.01, ***p < 0.001), and “ns” represents non-significance based on standard two-way ANOVA followed by Bonferroni multiple comparisons test

Relative water content (RWC) showed a significant interaction effect between watering treatment stage and SSP scenario (*p* = 0.023; Fig. 6). Under SSP1 and SSP3 scenarios, RWC decreased during the drought exposure stage compared with the pre-drought stage by 10.7% and 5.5%, respectively. During the post-rewatering recovery stage, RWC increased under SSP1, whereas it remained similar to the drought-stage level under SSP3. Under SSP5, RWC remained stable throughout the entire experimental period, showing no significant variation across watering treatment stages.

### Changes in morpho-anatomical traits according to watering treatment stages and SSP scenarios

Specific leaf area (SLA) and xylem vessel diameter (XD) showed no significant responses to watering treatment stage, SSP scenario, or their interaction (SLA: *p* = 0.146–0.260; Additional file 1: Fig. S1A, XD: *p* = 0.228–0.764; Additional file 1: Fig. S1B). Across the experimental period, SLA ranged from 80.1 to 118.6 cm^2^ g^−1^. XD showed a consistent pattern, with values ranging from 24.1 to 31.0 µm. No significant stage- or scenario-dependent variation was detected for either trait.

### Changes in biochemical status according to watering treatment stages and SSP scenarios

Total soluble sugar (TSS) concentration was not significantly affected by watering treatment stage, SSP scenario, or their interaction (*p* = 0.131–0.974; Additional file 1: Fig. S1C). Across all watering treatments, TSS concentration ranged from 2.87 to 7.09 mg g⁻^1^. Although TSS tended to be higher during the post-rewatering recovery stage than during the pre-drought and drought exposure stages, this difference was not statistically significant.

### Changes in stable carbon isotope composition ratios during the post-rewatering recovery stage

Stable carbon isotope composition (δ^13^C) of *D. trifidus* tended to decrease in all organs as the scenario shifted from lower to higher forcing (SSP1 → SSP5). Across all SSP scenarios, stems showed the lowest δ^13^C values (Additional file 1: Fig. S2). Under SSP1, which exhibited the highest organ-specific carbon isotope composition among the three SSP scenarios, δ^13^C values were 45.1‰ in leaves, 5.1‰ in stems, and 40.1‰ in fine roots. The smallest inter-organ variation in δ^13^C was observed under SSP3, with values of 1.7‰ in leaves, − 2.3‰ in stems, and 5.3‰ in fine roots. Meanwhile, the lowest organ-specific δ^13^C values were observed under SSP5, with values of − 1.3‰ in leaves, − 18.8‰ in stems, and − 13.3‰ in fine roots.

## Discussion

### Drought responses of *D. trifidus* under climate change-induced dry spell conditions

This study showed that drought exposure reduced photosynthetic performance and altered photosystem II (PS II) energy fluxes in *Dendropanax trifidus* seedlings. During the drought exposure stage, maximum quantum yield of PS II (*F*_*v*_/*F*_*m*_) decreased, whereas absorption flux per reaction center (ABS/RC), dissipated energy flux per reaction center (DI_*o*_/RC), electron transport flux per reaction center (ET_*o*_/RC), and electron flux to photosystem I acceptor side per reaction center (RE_*o*_/RC) increased. These responses suggest that drought induced photoprotective regulation aimed at mitigating further damage to the photosynthetic apparatus by inactivating part of the reaction centers and dissipating excess excitation energy as heat. Similar results have been reported in herbaceous and woody plants exposed to drought and heat stress [11, 20, 21]. In particular, the increases in ABS/RC and DI_*o*_/RC were more pronounced under SSP5, suggesting that when extreme climate conditions are combined with drought stress, excess excitation pressure on the photosystems increases, thereby enhancing the requirement for non-photochemical energy dissipation [11, 18, 19, 38, 39].

Drought exposure also strongly reduced leaf gas exchange. The concurrent decline in stomatal conductance (*g*_*s*_) and net photosynthetic rate (*P*_*n*_) under water deficit is also a typical gas exchange response observed in various C3 plants and woody seedlings [40–42]. In the present study, *P*_*n*_ and *g*_*s*_ decreased synchronously during the drought exposure stage across all SSP scenarios, demonstrating that stomatal regulation to minimize water loss operated strongly under water deficit. Notably, even considering this stomatal closure process for maintaining internal water status, the decline in *P*_*n*_ during drought exposure stage was far more pronounced than that in *g*_*s*_, causing intrinsic water use efficiency (WUE_*i*_) to decrease rather than increase.

This reduction in WUE_*i*_ indicates that the decline in photosynthetic rate during drought was not caused solely by the limitation of CO_2_ supply due to stomatal closure, suggesting substantial involvement of non-stomatal limitations in the reduction of *P*_*n*_. In particular, the substantial impairment of photosystem II photochemical reactions—directly confirmed in this study through chlorophyll fluorescence indicators such as the decrease in *F*_*v*_/*F*_*m*_ and the increases in ABS/RC and DI_*o*_/RC—provides empirical evidence supporting the actual occurrence of these non-stomatal limitations. These limitations likely involved a combination of the observed photochemical inhibition and biochemical limitations, such as the metabolic inactivation of various enzymes in the Calvin cycle, which together operated within the photosynthetic apparatus to drive the further reduction in P_n_ [43–46]. Furthermore, the absence of significant differences in WUE_*i*_ among SSP scenarios indicates that, under severe drought conditions, this inhibitory effect of water deficit on the photosynthetic apparatus was more dominant than the differences in growth environments among the SSP scenarios.

The leaf relative water content (RWC) results showed that leaf water status responded differentially among SSP scenarios, even under strong limitations on stomatal gas exchange. The decrease in RWC under SSP1 and SSP3 indicates that leaf tissue dehydration progressed during the drought exposure stage. In contrast, the relatively stable RWC observed under SSP5 suggests that the combined growth environment of elevated CO_2_ concentration and temperature may have partially buffered the decline in leaf water status caused by drought stress [14, 47].

The physiological limitations imposed on the photosynthetic apparatus and stomatal gas exchange during the drought exposure stage were associated with reduced growth and biomass accumulation in *D. trifidus*. Reduced photosynthetic activity can limit carbohydrate supply for tissue development and metabolic maintenance, thereby causing growth suppression [48–50]. Drought-induced reductions in height growth have also been reported in various broad-leaved species, including the evergreen hybrid *Eucalyptus urophylla* X *E. grandis* and the deciduous species *Quercus brantii*, *Quercus robur*, and *Fagus sylvatica* [48–50]. At the same time, the increase in the root-to-shoot ratio (R/S ratio) observed in drought-exposed *D. trifidus* indicates a shift in biomass allocation toward root growth. This resource allocation pattern represents a typical adaptive strategy for improving water acquisition by preferentially allocating carbohydrates to roots and promoting exploration of deeper soil layers [24, 25].

In contrast, morpho-anatomical traits such as specific leaf area (SLA) and xylem vessel diameter (XD) did not show clear changes in response to watering treatment stage or SSP scenario. This suggests that drought resistance in *D. trifidus* seedlings was associated with photoprotective adjustment, stomatal limitation, reduced carbon assimilation, and increased root allocation, while short-term morpho-anatomical changes were limited.

### Post-rewatering recovery dynamics of *D. trifidus* under climate change conditions

After rewatering, chlorophyll fluorescence responses in *D. trifidus* exhibited patterns opposite to those observed during the drought exposure stage. *F*_*v*_/*F*_*m*_ increased, whereas ABS/RC and DI_*o*_/RC, which had increased during drought, decreased. These responses indicate that the drought-induced photoprotective state maintained through excess energy dissipation and reaction center inactivation was alleviated, and that the photochemical function of PS II reaction centers was gradually restored [51]. In addition, the recovery of ET_*o*_/RC, representing energy flux transferred beyond the primary electron acceptor, and RE_*o*_/RC, reflecting energy flux reaching Photosystem I acceptors, suggested that the electron transport chain became progressively stabilized after drought relief alongside these positive changes in fluorescence indicators. This improvement in electron transport activity may have provided a physiological basis for the rapid rebound of *P*_*n*_ after rewatering by supporting the production of ATP and NADPH required for carbon fixation [52].

The recovery of gas exchange differed among SSP scenarios. After rewatering, both *P*_*n*_ and *g*_*s*_ increased under all SSP scenarios, but they exceeded their pre-drought values only under SSP3 and SSP5. Because CO_2_ concentration and air temperature were elevated simultaneously in this study, their independent effects cannot be separated. Nevertheless, the results suggest that the combined growth conditions under SSP3 and SSP5 were associated with photosynthetic recovery after rewatering. Under conditions of sufficient water availability, elevated air temperature can enhance photosynthesis-related enzymatic activity [53, 54], while elevated atmospheric CO_2_ concentration can improve photosynthetic efficiency and mitigate transpirational demand [14, 55]. Consequently, the compound future environments under SSP3 and SSP5, which combine elevated air temperature and atmospheric CO_2_ concentration, likely exerted a synergistic effect, accelerating the post-rewatering recovery stage of stomatal opening and metabolic activity to drive the observed responses.

Leaf gas exchange responses and RWC are distinct physiological indicators, but they are closely linked through stomatal regulation [56–58]. Therefore, evaluating post-rewatering recovery requires consideration not only of the restoration of gas exchange but also of whether tissue water status recovered concurrently. In this study, the RWC dynamics during the post-rewatering recovery stage did not fully correspond to the recovery patterns of gas exchange. Under SSP1, RWC tended to gradually recover toward the pre-drought level, whereas under SSP3, RWC remained at a low level after rewatering, similar to that observed during the drought exposure stage. This suggests that, under SSP3, gas exchange resumed through stomatal reopening as in the other SSP scenarios, whereas the recovery of RWC may have been delayed. In contrast, RWC under SSP5 remained stable throughout the experiment. Overall, the RWC dynamics during the post-rewatering recovery stage showed a nonlinear relationship with the numerical recovery of *P*_*n*_ and *g*_*s*_. These results indicate that the relationship between gas exchange recovery and internal water balance may differ depending on the compound climate environment represented by each SSP scenario, reflecting plant water use patterns that account for scenario-specific growth environments.

Growth performance during the recovery stage was consistent with the observed recovery of gas exchange. The root-to-shoot ratio decreased after rewatering, following its increase during drought exposure. This pattern suggests that biomass allocation shifted from belowground investment during drought toward greater aboveground development after water resupply [26, 27]. In particular, the increase in net photosynthetic rate during the post-rewatering recovery stage compared with the drought exposure stage may have supplied sufficient carbohydrates to support aboveground growth [48–50]. This reversal of resource allocation is consistent with the significantly higher RRG and RHG observed during the post-rewatering recovery stage than during the drought exposure stage.

These results suggest that post-rewatering recovery in *D. trifidus* involved the relaxation of photoprotective regulation, partial restoration of PS II function, recovery of gas exchange, and redistribution of biomass toward aboveground organs. The stronger recovery of *P*_*n*_ and *g*_*s*_ under SSP3 and SSP5 further suggests that elevated CO_2_ concentration and warming, under the tested conditions, can modify the recovery trajectory of *D. trifidus* after drought. However, these results should be interpreted with caution because key hydraulic indicators, such as leaf or stem water potential, hydraulic conductance, and leaf/cuticle anatomical traits were not measured. Future studies should include hydraulic measurements to clarify how water transport, tissue water status, and photosynthetic recovery are coordinated during drought and post-rewatering recovery.

## Conclusions

This study examined drought resistance and post-rewatering recovery responses of *Dendropanax trifidus* seedlings under SSP-based climate scenarios combining elevated CO_2_ concentration and warming. During drought exposure stage, *D. trifidus* showed reduced photosynthetic carbon assimilation, strong stomatal limitation, increased photoprotective energy dissipation, and greater biomass allocation toward belowground organs. These responses suggest that drought resistance in this species was associated with photosystem protection, reduced water loss, and root-oriented biomass allocation rather than the maintenance of photosynthetic carbon gain.

After rewatering, *D. trifidus* showed partial recovery of photosynthetic function, gas exchange, and growth. The decrease in absorption flux per reaction center (ABS/RC) and dissipated energy flux per reaction center (DI_*o*_/RC), together with the increase in maximum quantum yield of photosystem II (*F*_*v*_/*F*_*m*_), suggests that the drought-induced photoprotective state was alleviated during recovery. Biomass allocation also shifted away from root dominance, indicating renewed aboveground growth after water resupply. However, the extent of recovery differed among SSP scenarios. Under SSP3 and SSP5, net photosynthetic rate (*P*_*n*_) and stomatal conductance (*g*_*s*_) exceeded pre-drought stage, whereas under SSP1 they remained below pre-drought stage levels. Although the independent effects of CO_2_ concentration and temperature could not be separated, these results suggest that the compound environments represented by SSP3 and SSP5 influenced post-rewatering photosynthetic recovery under the conditions tested in this study. In contrast, specific leaf area (SLA) and xylem vessel diameter (XD) did not change significantly across watering treatment stages or SSP scenarios. This indicates that drought and recovery responses in *D. trifidus* were expressed mainly through physiological regulation and biomass allocation adjustments rather than structural modification.

These findings provide baseline information for understanding the ecological adaptability of *D. trifidus* under future climate scenarios and may offer practical insights for species management and stand establishment strategies in the Korean Peninsula. However, gas exchange parameters were derived from only three seedlings per scenario, and carbon isotope analysis was conducted using only one seedling per scenario. In addition, key hydraulic indicators, such as leaf or stem water potential, were not measured. Future studies should incorporate greater replication and direct hydraulic measurements to clarify how water transport, tissue water status, carbon allocation, and photosynthetic recovery are coordinated during drought and post-rewatering recovery.

## Methods

### Experimental setup and plant materials

Future climate simulation experiments were conducted from May 10 to June 30, 2024, in Soil Plant Daylit System (SPDS) chambers (EGC Corp., Chagrin Falls, USA) at the Climate Change Education Center of Korea National University of Agriculture and Fisheries, Jeonju, Republic of Korea. Each chamber measured 3 m in length, 4 m in width, and 3 m in height, and the chambers were spaced 10 m apart. The SPDS chamber is a precision environmental control system covered with polymethyl methacrylate (PMMA) panels, allowing seasonal light intensity and spectral characteristics to be reproduced more realistically than in conventional artificial chambers (Additional file 2: Fig. S3).

**Fig. 7:**
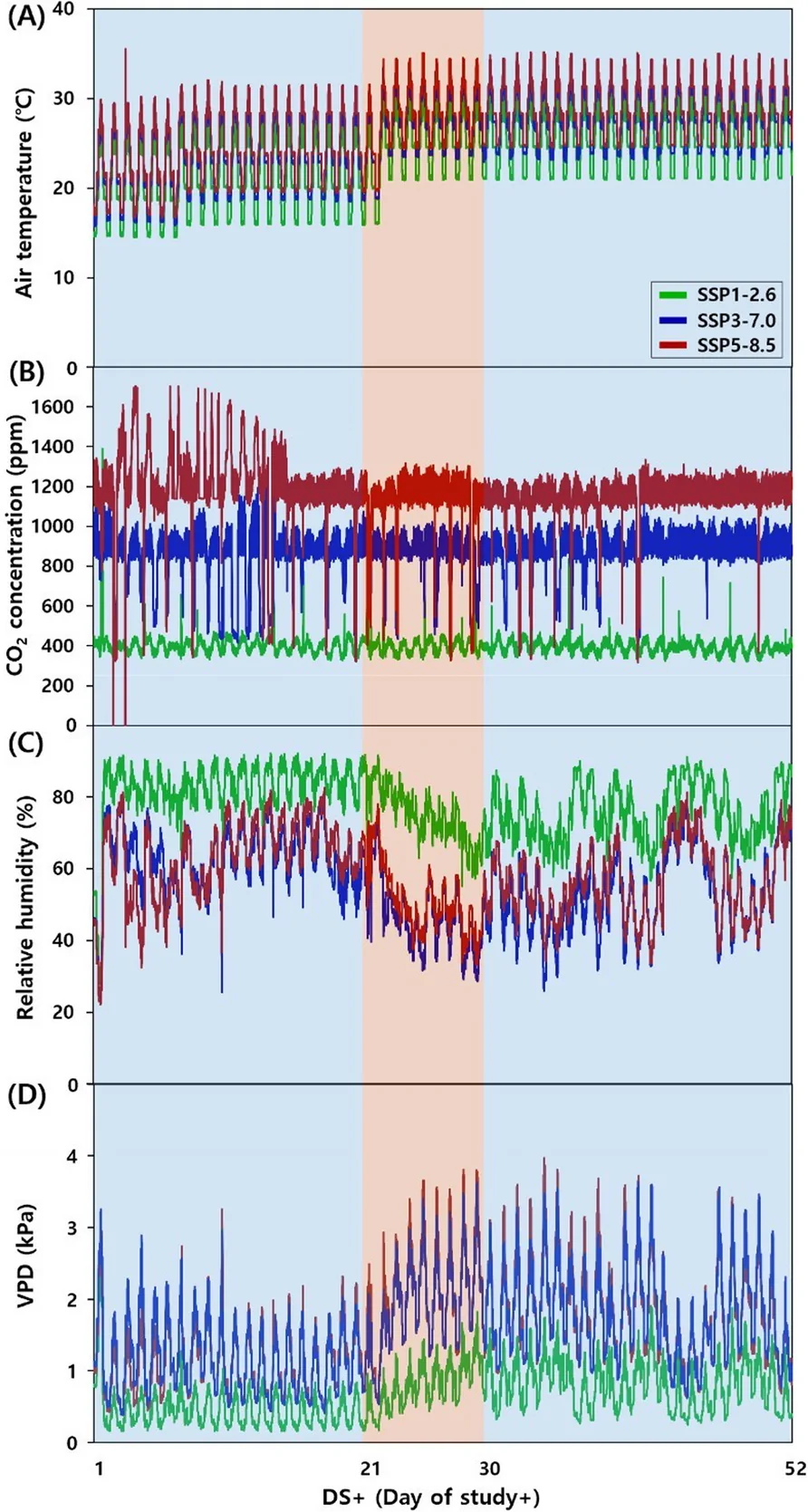
SPDS (Soil Plant Daylit System) chamber experimental conditions with scenario-specific time series of environmental factors. **A** Air temperature (°C), **B** atmospheric CO_2_ concentration (ppm), **C** relative humidity (%), **D** vapor pressure deficit (VPD) within the SPDS chambers from day 1 to day 52. Blue shaded areas represent pre-drought exposure and post-rewatering recovery stages, while light red shaded areas indicate the drought exposure stage

This study simulated climatic conditions corresponding to three representative socioeconomic pathway (SSP) scenarios: SSP1-2.6 (hereafter SSP1), SSP3-7.0 (hereafter SSP3), and SSP5-8.5 (hereafter SSP5). Based on projected atmospheric CO_2_ concentrations from the simplified climate-carbon cycle model MAGICC 7.0 [59] and dynamically downscaled UKESM1 temperature projections for East Asia for the late twenty-first century (2080–2100) [37], SSP1 was set to 440 ppm CO_2_ and + 2.3 °C relative to the May–June mean temperature in Jeonju, SSP3 to 870 ppm CO_2_ and + 5.4 °C, and SSP5 to 1140 ppm CO_2_ and + 6.3 °C. Because each SSP scenario simultaneously prescribed elevated CO_2_ concentration and increased temperature, these two factors were inherently coupled within each scenario by design. Therefore, the responses reported in this study reflect the integrated effects of each compound climate condition rather than the independent effects of CO_2_ concentration or temperature, and no attempt was made to separate these two factors. The MAGICC 7.0 projections were refined using a multistep downscaling framework to ensure spatial consistency between global CO_2_ trends and regional temperature increments [59]. The global UKESM1 projections used for chamber temperature settings were dynamically downscaled to a 25 km resolution over East Asia using five regional climate models, including HadGEM3-RA, and were subsequently refined into 1 km high-resolution Korea-specific scenarios using the PRIDE (Parameter-elevation Regressions on Independent Slopes Model) statistical downscaling method [37]. Each SSP scenario was programmed into three randomly selected SPDS chambers, resulting in a total of nine chambers, together with the 1994–2023 mean relative humidity in Jeonju based on Korea Meteorological Administration data (60% in May and 70% in June) (Additional file 3: Table S1). To ensure that each chamber consistently maintained the target conditions for its assigned SSP scenario, air temperature, CO_2_ concentration, and relative humidity were measured and recorded at 5 min intervals throughout the experiment using an RBF185L temperature sensor (Pyromation Inc., Fort Wayne, USA), a GMW86P CO_2_ transmitter (Vaisala, Vantaa, Finland), and a HMW82 humidity transmitter (Vaisala, Vantaa, Finland), respectively (Fig. 7A–C). Vapor pressure deficit (VPD) was calculated using the measured relative humidity and air temperature data (Fig. 7D).

Two-year-old containerized seedlings of *Dendropanax trifidus* (Thunb.) Makino ex H. Hara produced at the Boseong National Forest Nursery of the Western Regional Office of Forest Service were used as plant materials. On April 8, 2024, a nursery substrate composed of 25% coco peat, 24.8% peat moss, 25% perlite, 24% vermiculite, and 2% zeolite (Nongkyung, Jincheon, Republic of Korea) was uniformly filled into 3.1 L containers at 730–740 g per container using a digital balance, and one seedling was transplanted into the center of each container. The seedlings were acclimated for approximately 1 month before the experiment, and seedlings with uniform height and root collar diameter (RCD) were selected immediately before the experiment (Additional file 4: Table S2).

Six uniform *D. trifidus* seedlings were placed in each of the nine SPDS chambers, resulting in 18 seedlings per SSP scenario and 54 seedlings in total. Seedlings in each chamber were sequentially exposed to three watering treatment stages based on the day of study (DS): the pre-drought stage (DS + 1 to DS + 20), drought exposure stage (DS + 21 to DS + 30), and post-rewatering recovery stage (DS + 31 to DS + 52). During the pre-drought stage, each seedling received 600 mL of water every 3 days. During the drought exposure stage, irrigation was withheld for 10 days to simulate drought associated with future climate change. During the post-rewatering recovery stage, irrigation was resumed at the same frequency and amount as in the pre-drought stage. The duration of the drought exposure stage was determined based on a critical physiological stress threshold that induces maximal adaptive responses without causing irreversible mortality [60, 61]. Accordingly, the drought exposure stage was conducted while visually monitoring seedling wilting, and the termination criterion was set as the point at which maximum quantum yield of photosystem II (*F*_*v*_/*F*_*m*)_ declined below 0.5, a range indicating severe stress [60, 61]. On the 10th day of the drought exposure stage, *F*_*v*_/*F*_*m*_ values in all experimental groups were confirmed to have decreased below 0.5; the drought treatment was then terminated, and rewatering was initiated. The post-rewatering recovery stage was maintained for 22 days, longer than the drought exposure stage, to allow clear observation of physiological and morphological recovery.

Before the watering treatments and SSP scenarios were imposed, the six seedlings in each chamber were assigned to harvest schedules according to watering treatment stage, and each seedling was harvested only once during the experiment. Seedlings harvested at the pre-drought stage and drought exposure stage were used to measure variables requiring destructive harvest, including total biomass, R/S ratio, leaf relative water content (RWC), specific leaf area (SLA), xylem vessel diameter (XD), and total soluble sugar (TSS) concentration. In contrast, seedlings maintained until the post-rewatering recovery stage were used for repeated non-destructive measurements throughout the experiment, including height, root collar diameter, net photosynthetic rate (*P*_*n*_), stomatal conductance (*g*_*s*_), intrinsic water use efficiency (WUE_*i*_), and chlorophyll fluorescence. These seedlings were finally harvested at the end of the post-rewatering recovery stage to measure total biomass, R/S ratio, RWC, SLA, XD, and TSS.

**Fig. 8:**
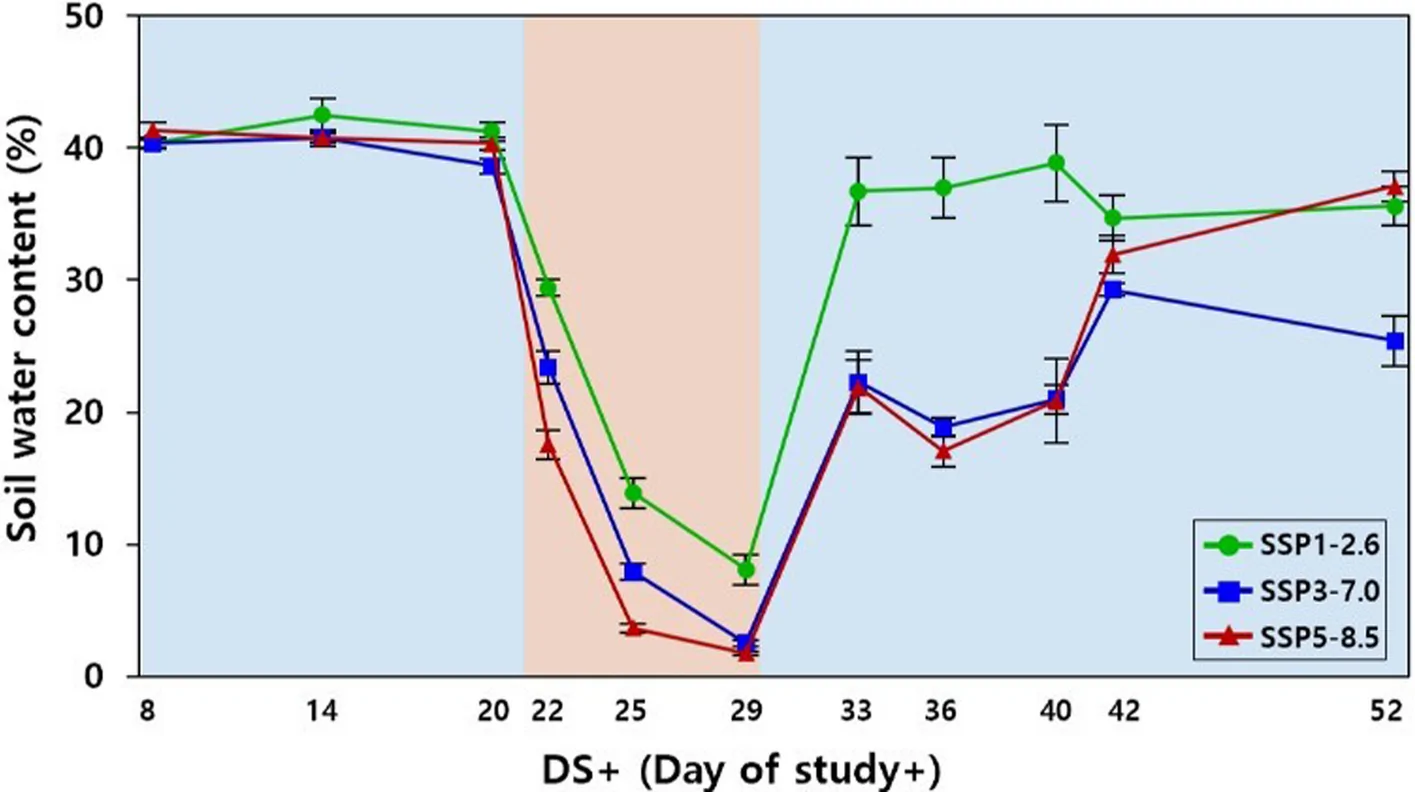
Scenario-specific fluctuations in soil moisture content during the experimental period. Areas shaded in blue represent pre-drought and post-rewatering recovery stages, while those in light red indicate the drought exposure stage. Vertical bars indicate the standard error of the mean (*n* = 3)

Soil water content was monitored using a soil moisture meter (WT-1000N, RF Sensor, Seoul, Republic of Korea). Except for the final measurement, measurements were conducted at 6-day intervals during the pre-drought stage and at 3–4-day intervals during the drought exposure and post-rewatering recovery stages. Two seedlings per chamber were randomly selected, resulting in six seedlings per SSP scenario and 18 seedlings in total, and the same seedlings were repeatedly monitored throughout the experiment (Fig. 8).

### Growth performance

Two seedlings per chamber, corresponding to six seedlings per SSP scenario and 18 seedlings in total, were designated for height and root collar diameter measurements throughout the experiment. Height and root collar diameter were measured at the end of each watering treatment stage using a folding ruler and a digital vernier caliper, respectively. Seedling height was measured as the sum of (1) the pre-measured distance between the soil surface and the container rim and (2) the height from the container rim to the uppermost terminal bud, thereby ensuring consistency in the measurement reference point. For root collar diameter, a reference line was marked with a permanent marker 1 cm above the soil surface and repeatedly measured to a precision of 0.01 cm. Height and root collar diameter increments were converted to daily growth rates by dividing them by the duration of each watering treatment stage. Relative daily height growth rate (RHG) and relative daily root collar diameter growth rate (RRG) were calculated from repeated measurements of height and root collar diameter on the same seedlings. These seedlings used for repeated non-destructive measurements were finally harvested after the post-rewatering recovery stage.

For biomass measurement, all seedlings used in the experiment were harvested once at one of the three watering treatment stages. At the end of each watering treatment stage, two seedlings per chamber, corresponding to six seedlings per SSP scenario and 18 seedlings in total, were harvested for biomass measurement. After sample collection for morpho-anatomical and biochemical analyses, harvested seedlings were separated into aboveground (stem) and belowground (root) components. Each plant component was dried at 65 °C for 72 h and weighed using a digital balance. The root-to-shoot ratio (R/S ratio) was then calculated from the measured dry weights.

### Physiological states

To examine changes in photosynthetic electron transport processes according to SSP scenario and watering treatment stage, chlorophyll fluorescence was measured in five seedlings per SSP scenario. Seedlings selected for measurement were randomly chosen before treatment imposition: two seedlings from each of two chambers and one seedling from the remaining chamber. The same five seedlings were repeatedly measured throughout the experiment. Measurements were conducted using a Plant Efficiency Analyzer (PEA; Hansatech Instruments Ltd., King’s Lynn, England) and an OS30p + fluorometer (Opti-Sciences Inc., New Hampshire, USA) at five measurement stages: early pre-drought stage (DS + 14), late pre-drought stage (DS + 20), mid-drought stage (DS + 26), peak-drought stage (DS + 29), and post-rewatering recovery stage (DS + 43). The PEA was used at the early pre-drought stage (DS + 14) and late pre-drought stage (DS + 20). However, because of PEA malfunction after the late pre-drought stage (DS + 20), measurements at the mid-drought stage (DS + 26), peak-drought stage (DS + 29), and post-rewatering recovery stage (DS + 43) were conducted using the OS30p + . Because the PEA malfunction occurred during the experiment, duplicate cross-calibration measurements between the PEA and OS30p + could not be performed. To reduce and examine the potential influence of instrument-specific calculation procedures, fluorescence parameters obtained with the OS30p + at DS + 26, DS + 29, and DS + 43 were recalculated using the PEA-based formulas [62], while fluorescence parameters obtained with the PEA at DS + 14 and DS + 20 were also recalculated using the OS30p + based formulas [63]. This comparison showed that the absolute values differed slightly depending on the formula set, with PEA-based values tending to be lower than OS30p + -based values. However, the direction of increase or decrease across measurement stages was consistent between the two calculation approaches. This consistency is important because OJIP-derived fluorescence parameters are generally used to evaluate relative changes across treatment or stress stages rather than to interpret absolute values at a single time point. Therefore, in this study, chlorophyll fluorescence results were interpreted primarily in terms of stage-dependent changes during drought exposure and post-rewatering recovery, rather than absolute parameter values. In addition, comparisons among the mid-drought stage (DS + 26), peak-drought stage (DS + 29), and post-rewatering recovery stage (DS + 43) were based on measurements obtained using the same OS30p + instrument; thus, comparisons within this period were considered unaffected by instrument replacement. For chlorophyll fluorescence measurements, the uppermost healthy leaf was selected and dark-adapted for 20 min before being exposed to 3500 μmol m⁻2 s⁻1 light for 1 s. Fluorescence intensity was recorded at 50 μs (O step), 300 μs (K step), 2 ms (J step), 30 ms (I step), and 500 ms (P step). Technical fluorescence parameters, specific energy fluxes, and performance indices were calculated using OJIP analysis and the JIP-test approach (Table 1).

**Table 1:** Mathematical expressions and definitions of chlorophyll fluorescence parameters based on [62–64]

Abbreviation	Mathematical expressions	Description
*Technical fluorescence parameters*		
*F*_*o*_	${F}_{o}$	Initial fluorescence at 50 μs from dark-adapted tissue
*F*_*m*_	${F}_{m}$	The maximal fluorescence emission obtained in the curve
*F*_*v*_	${F}_{m}-{F}_{o}$	Maximal variable fluorescence
*F*_*v*_/*F*_*m*_	$({F}_{m}-{F}_{o})/{F}_{m}$	Probability that an absorbed photon leads to reduction further than *Q*_*A*_^−^
*Specific energy fluxes (per active photosystem II reaction center)*		
ABS/RC	${(M}_{o}/{V}_{J})/({F}_{v}/{F}_{m})$	Absorption flux per reaction center
TR_*o*_/RC	${M}_{o}/{V}_{J}$	Trapped energy flux per reaction center at *t* = 0
ET_*o*_/RC	${(M}_{o}/{V}_{J})- {M}_{o}$	Electron transport flux from *Q*_*A*_ to *Q*_*B*_ per reaction center at *t* = 0
DI_*o*_/RC	$\mathrm{A}\mathrm{B}\mathrm{S}/\mathrm{R}\mathrm{C}-{\mathrm{T}\mathrm{R}}_{o}/\mathrm{R}\mathrm{C}$	Energy dissipation flux per reaction center at *t* = 0
RE_*o*_/RC	${(M}_{o}/{V}_{J})(1-{V}_{I})$	Electron transport flux until photosystem I acceptors per reaction center at* t* = 0
*Performance index*		
PI_abs_	$\frac{1-({F}_{o}/{F}_{m})}{{M}_{o}/{V}_{J}} \cdot \frac{{F}_{m}-{F}_{o}}{{F}_{o}}\cdot \frac{1-{V}_{J}}{{V}_{J}}$	Performance index on absorption basis

*M*_*o*_ : approximated initial slope (in ms^–1^) of the fluorescence transient *V* = *f*(*t*), *V*_*j*_ : relative variable fluorescence at the *J*-step, *V*_*I*_ : relative variable fluorescence at the *I*-step, *Q*_*A*_ : primary plastoquinone acceptor, *Q*_*B*_ : secondary quinone

At the end of each watering treatment stage, one seedling per chamber, corresponding to three seedlings per SSP scenario, was selected to measure net photosynthetic rate (*P*_*n*_). Measurements were conducted using a portable photosynthesis system (LI-6400XT; LI-COR, Inc., Lincoln, NE, USA), and the same seedlings were repeatedly measured throughout the experiment. During measurement, the CO_2_ concentration and temperature inside the leaf cuvette were set to match the CO_2_ concentration and temperature of the chamber in which each seedling was placed. Air flow rate, relative humidity, and photosynthetic photon flux density (PPFD) were maintained at 500 μmol s⁻1, 60%, and 1200 μmol m^⁻2^ s^⁻1^, respectively. Using directly measured gas exchange parameters from the LI-6400XT, stomatal conductance to water vapor (*g*_*s*_) and intrinsic water use efficiency (WUE_*i*_) were calculated following the equations presented in Table 2 to gain further comprehension of the target seedlings' hydraulic status and water stress levels.

**Table 2:** Equations used for calculating stomatal conductance and intrinsic water use efficiency

Physiological parameter	Equation
Stomatal conductance (*g*_*s*_)	${g}_{s}={g}_{0}+1.6(1+\frac{{g}_{1}}{\sqrt{D}})\frac{{P}_{n}}{{C}_{a}}$
Intrinsic water use efficiency (WUE_*i*_)	${\mathrm{W}\mathrm{U}\mathrm{E}}_{i}=\frac{{P}_{n}}{{g}_{s}}$

*g*_*s*_ is the stomatal conductance to water vapor in mol m^−2^ s^−1^, *g*_0_ is the residual stomatal conductance when net leaf photosynthesis is 0 (μmol m^−2^ s^−1^), *g*_1_ is a dimensional parameter indicating plant’s marginal water cost of carbon gain, D is the vapor pressure deficit at the leaf surface in kPa, *P*_*n*_ is the net photosynthesis rate (μmol m⁻^2^ s⁻^1^), *C*_*a*_ is the concentration of CO_2_ at the leaf surface (μmol mol⁻^1^), 1.6 is the ratio of water vapor diffusivity relative to CO_2_ in the air, and WUE_*i*_ is the intrinsic water use efficiency (μmol mol⁻^1^). The *g*_*s*_ equation was retrieved from [65, 66]. In this equation, *g*_0_ was set to 0.01 based on the value reported for evergreen broad-leaved tree species [67], and *g*_1_ was parameterized based on 4.12 reported for temperate evergreen broad-leaved tree species [68], following the degree of agreement demonstrated between empirical and equation-based outputs (*r*^2^ = 0.61) [69]. The WUE_*i*_ equation was obtained from [70]

Leaf relative water content (RWC) was calculated using leaves from the same seedlings used for SLA measurement at each watering treatment stage. Fresh weight (FW) was measured immediately after sampling, after which leaves were immersed in distilled water overnight to reach full hydration. After removing surface moisture, turgid weight (TW) was measured, and the leaves were then dried at 65 °C for 72 h to determine dry weight (DW). RWC was calculated as follows:

1 $$\mathrm{RWC}\ (\%)\ =\ (\mathrm{FW}- \mathrm{DW})/ (\mathrm{TW}-\mathrm{DW})\times 100$$

### Morpho-anatomical traits

To evaluate morpho-anatomical traits according to watering treatment stage and SSP scenario, specific leaf area (SLA) and xylem vessel diameter (XD) were measured using seedlings independently harvested at the end of each watering treatment stage. For SLA measurement, one harvested seedling per chamber was randomly selected, resulting in three seedlings per SSP scenario. Thus, SLA was analyzed based on chamber-level observations. Three fully expanded healthy upper leaves were collected from each selected seedling between 14:00 and 16:00. Following the method of [71], collected leaves were separated into lamina and petiole, and the laminae were scanned using a scanner (Epson Perfection V800 Photo, Epson, Nagano, Japan). Leaf area was measured using ImageJ (Wayne Rasband/NIH, Bethesda, MD, USA). The leaves were then dried at 65 °C for 72 h, weighed using a precision electronic balance, and used to calculate SLA.

XD was measured using the same seedlings used for SLA measurement. Cross sections were prepared from the middle portion of the seedling stem using the free-hand sectioning method [72]. Sections were stained with toluidine blue, dehydrated with 80% ethanol, and mounted on microscope slides. The prepared slides were photographed at 200 × magnification using a Nikon optical microscope (Nikon, Japan) equipped with LAS X imaging software (Leica Microsystems Ltd., Wetzlar, Germany). Stem cross-section images were exported to ImageJ, and the maximum and minimum diameters of vessels and their mean values were measured. When it was difficult to distinguish axial parenchyma from xylem fibers in small vessels, the mean diameter of the 25 largest vessels, which play an important role in water transport, was used as the representative vessel diameter for each experimental group. As with SLA, XD was measured using seedlings independently harvested at each watering treatment stage.

### Biochemical status

At the end of each watering treatment stage, one seedling per chamber was randomly selected from the six harvested seedlings, resulting in three seedlings per SSP scenario, and total soluble sugar (TSS) concentration was analyzed using the anthrone-sulfuric acid method [73]. Because TSS was measured in one seedling per chamber, this single measurement was used directly as the chamber-level observation. A 10 cm stem segment above the permanent marker used as the root collar diameter reference point was excised. To prevent enzymatic sugar degradation, samples were heated in a microwave for 1 min 30 s within 3 h after collection. The samples were then dried at 65 °C for 72 h and ground. A 50 mg powdered sample was mixed with 5 mL of 80% ethanol and heated in a water bath at 80–85 °C for 30 min. After cooling, the mixture was centrifuged at 3000 rpm for 10 min, and the supernatant was collected in a 50 mL beaker. The extraction procedure from ethanol addition to supernatant collection was repeated once, and the collected supernatant was brought to 50 mL with 80% ethanol to prepare the TSS extract. Then, 0.5 mL of extract and 4.5 mL of distilled water were mixed in a glass tube and kept on ice. Anthrone reagent (10 mL) was slowly added while stirring with a glass rod, and the mixture was heated in boiling water for 7 min 30 s. Immediately after heating, the glass tubes were placed on ice and completely cooled. Absorbance was measured within 1 h at 620 nm using a spectrophotometer (UVmini-1240, Shimadzu Co., Kyoto, Japan). A standard calibration curve was prepared using dextrose (Dextrose 98.0%, Samchun, Pyeongtaek, Republic of Korea) for TSS quantification.

### Stable carbon isotope composition analysis

To evaluate the distribution of photosynthetic products during the post-rewatering recovery stage, ^13^CO_2_ pulse labeling was conducted on the 16th day after the onset of the post-rewatering recovery stage (DS + 46). During the early post-rewatering recovery stage (days 1–3), metabolic fluxes are unstable, and newly fixed carbon tends to be preferentially allocated to tissue repair and maintenance respiration rather than structural growth [74, 75]. Therefore, the 16th day was selected as the optimal time point for observing whole-plant recovery strategies, when photosynthesis was expected to have stabilized and carbon allocation for new structural growth was likely to be concentrated.

The chamber for ^13^CO_2_ pulse labeling was custom-built in the laboratory. The chamber frame consisted of stainless steel pipes assembled inside an acrylic box measuring 0.5 m in width, 0.3 m in depth, and 0.9 m in height. An air circulation fan and instruments for measuring CO_2_ concentration and temperature during pulse labeling (MI70 handheld meter with GMP343 probe; Vaisala, Vantaa, Finland) were installed on the pipe frame. Ice packs were placed on both sides of the acrylic box to prevent rapid temperature increases during pulse labeling. One seedling from each SSP scenario was selected and placed together in the _13_CO_2_ pulse-labeling chamber. The chamber was covered with 0.01 mm thick plastic film and sealed to the acrylic box with transparent tape. To maintain stable environmental conditions during labeling, the labeling chamber was placed inside a large chamber, and the ambient temperature was maintained at 25 °C. After the CO_2_ concentration inside the labeling chamber stabilized, 150 mL of ^13^CO_2_ (99 atom% 13C, Cambridge Isotope Laboratories, Inc., Tewksbury, MA, USA) was injected into a tube at the top center of the chamber using a syringe, and pulse labeling was conducted for 60 min. After labeling, each seedling was returned to its original chamber and harvested 7 days later for carbon isotope analysis. Harvested seedlings were thoroughly washed with running water to remove soil and then rinsed with distilled water. They were separated into leaves, stems, and fine roots (< 2 mm in diameter). Each tissue was dried at 65 °C for 72 h, ground, and stored at − 20 °C until analysis.

Carbon isotope composition of the ground samples was analyzed at the National Instrumentation Center for Environmental Management (NICEM) using an elemental analyzer (Elementar, Germany) coupled to a continuous-flow isotope ratio mass spectrometer (IsoPrime Vision-EA, UK). Carbon isotope ratios were expressed as delta (*δ*) values (‰) relative to the Pee Dee Belemnite (PDB) standard using Eq. 2. In this equation, *R*_sample_ represents the ^13^C/^12^C ratio of each analyzed plant tissue, and *R*_standard_ represents the ^13^C/^12^C ratio of PDB (0.0112372) [76]. Because only one seedling per SSP scenario was analyzed (n = 1), atom% excess values were not calculated. The δ^13^C values were used only for qualitative and exploratory comparisons of organ-level carbon allocation patterns among watering treatments and SSP scenarios, without inferential statistical analysis.

2 $$\delta\ (\%_0)\ =\ \lbrack(R_{sample}/R_{standard})-1\rbrack\ \times\ 1000$$

### Statistical analysis

Statistical analyses were performed to evaluate the responses of *D. trifidus* according to watering treatment stage and SSP scenario. Prior to analysis, all data were tested for normality of residuals and homogeneity of variance (Additional file 5: Table S3). In this study, SSP scenarios were applied at the level of the SPDS chamber rather than at the level of individual seedlings. Therefore, when testing SSP scenario effects, the chamber was defined as the experimental unit, with three chambers included in each SSP scenario (*n* = 3 chambers per SSP scenario). Individual seedlings or leaves measured within each chamber were treated as subsamples rather than as independent experimental replicates. When multiple seedlings or leaves were measured within a chamber, these values were averaged and used as the representative value for that chamber. In contrast, for variables measured in only one seedling per chamber, such as *P*_*n*_ and TSS, the single measurement was used directly as the chamber-level observation. Accordingly, all inferential statistical analyses were based on chamber-level values rather than treating individual seedlings or leaves as independent replicates. For detailed information on the statistical analyses, please refer to Additional file 5: Table S3.

A two-way repeated-measures ANOVA (two-way RM ANOVA) was performed for chlorophyll fluorescence parameters repeatedly measured on the same seedlings (*F*_*v*_/*F*_*m*_, PI_abs_, and the other parameters listed in Table 1), net photosynthetic rate (*P*_*n*_), and growth performance indicators (RHG and RRG) calculated from repeated measurements of height and root collar diameter. For each model, the corresponding chlorophyll fluorescence parameter, *P*_*n*_, *g*_*s*_, WUE_*i*_, RHG, or RRG was treated as the dependent variable. SSP scenario was included as the between-subject factor in all two-way RM ANOVA models, whereas measurement stage or watering treatment stage was included as the within-subject factor, depending on the response variable. For chlorophyll fluorescence, measurements obtained from five seedlings per SSP scenario (two seedlings from each of two chambers and one seedling from the remaining chamber) were averaged within each chamber to derive chamber-level values before analysis. The five measurement stages—early pre-drought stage (DS + 14), late pre-drought stage (DS + 20), mid-drought stage (DS + 26), peak-drought stage (DS + 29), and post-rewatering recovery stage (DS + 43)—were included as the levels of the within-subject factor. For *P*_*n*_, one seedling per chamber was repeatedly measured at the end of each watering treatment stage; thus, the single measurement from each chamber was used directly as the chamber-level repeated observation. Watering treatment stage comprised three levels: pre-drought stage, drought exposure stage, and post-rewatering recovery stage. *g*_*s*_ and WUE_*i*_ were calculated from *P*_*n*_ and were analyzed using the same two-way RM ANOVA structure. For RHG and RRG, drought exposure stage and post-rewatering recovery stage were included as the two levels of the within-subject factor. Mauchly’s test of sphericity was conducted for models with three or more within-subject levels to assess the sphericity assumption.

A standard two-way ANOVA was performed for total biomass, R/S ratio, RWC, SLA, XD, and TSS, which were measured using different seedlings harvested at each watering treatment stage. Because different seedlings were harvested at each watering treatment stage, treatment stage differences for these variables were interpreted as differences among chamber-level observations based on separately harvested seedlings, rather than as temporal changes in the same seedlings. In this analysis, SSP scenario and watering treatment stage were included as independent variables, with watering treatment stage comprising three levels: pre-drought stage, drought exposure stage, and post-rewatering recovery stage.

For both the two-way RM ANOVA and the standard two-way ANOVA, when the interaction was not significant, the main effects of each factor were examined, and significant main effects were further compared among factor levels using Bonferroni-adjusted post hoc multiple comparisons. Statistical significance was determined at *p* < 0.05.

## Supplementary Information

Additional file 1: Fig. S1-S2. Fig. S1, SLA, XD, and TSS concentration across watering treatment stages and SSP scenarios. Fig. S2, Carbon isotopic composition (δ^13^C; ‰) of *Dendropanax trifidus* leaves, stems, and fine roots.

Additional file 2: Fig. S3. SPDS chambers used in the experiment.

Additional file 3: Table S1. Scenario-specific environmental control parameters applied in nine SPDS chambers.

Additional file 4: Table S2. Initial growth parameters of *Dendropanax trifidus* seedlings before SSP scenario and watering treatments.

Additional file 5: Table S3. Summary of statistical model specifications for all response variables.

## Data Availability

All data generated or analyzed during this study are included in this published article and its supplementary information files. Further inquiries can be directed to the corresponding authors.

## Abbreviations

ABS/RC: Absorption flux per reaction center
DI_*o*_/RC: Dissipated energy flux per reaction center at *t* = 0
DS: Day of study
ET_*o*_/RC: Electron transport flux from Q_A_ to Q_B_ per reaction center at *t* = 0
*F*_*v*_/*F*_*m*_: Maximum quantum yield of photosystem II
*g*_*s*_: Stomatal conductance to water vapor
PI_abs_: Performance index on an absorption basis
P_n_: Net photosynthetic rate
R/S ratio: Root-to-shoot ratio
RE_*o*_/RC: Electron transport flux to photosystem I acceptors per reaction center at *t* = 0
RHG: Relative height growth rate
RRG: Relative root collar diameter growth rate
RWC: Relative water content
SLA: Specific leaf area
SSP: Shared socioeconomic pathway
TR_*o*_/RC: Trapped energy flux per reaction center at *t* = 0
TSS: Total soluble sugar
WUE_*i*_: Intrinsic water use efficiency
XD: Xylem vessel diameter
δ^13^C: Stable carbon isotopic composition
