# Supplementary material for: Drought resistance and post-rewatering recovery of Dendropanax trifidus under elevated CO2 and temperature are mediated by photoprotective regulation and carbon reallocation
Source: BMC Biol. 2026 Aug 14;24:237. doi: 10.1186/s12915-026-02707-3 (PMC13760885; doi:10.1186/s12915-026-02707-3)
Supplement: Supplementary file 1 [file 12915_2026_2707_MOESM1_ESM.docx]

**Fig. S1** SLA, XD, and TSS concentration across watering treatment stages and SSP scenarios. The figures show (A) specific leaf area (SLA), (B) xylem vessel diameter (XD), and (C) total soluble sugar concentration (TSS) of *Dendropanax trifidus* across watering treatment stages and SSP scenarios. The three watering treatment stages were the pre-drought stage (Pre-drought in the figures), drought exposure stage (Drought in the figures), and post-rewatering recovery stage (Recovery in the figures). Vertical bars indicate the standard error of the mean (n = 3). Asterisks indicate statistical significance (**p* < 0.05, ***p* < 0.01, ****p* < 0.001), and “ns” represents non-significance based on standard two-way ANOVA followed by Bonferroni multiple comparisons test.

**Fig. S2** Carbon isotopic composition (δ^13^C; ‰) of *Dendropanax trifidus* leaves, stems, and fine roots. Values measured under the post-rewatering recovery stage across different SSP scenarios.
