# Supplementary material for: Drought resistance and post-rewatering recovery of Dendropanax trifidus under elevated CO2 and temperature are mediated by photoprotective regulation and carbon reallocation
Source: BMC Biol. 2026 Aug 14;24:237. doi: 10.1186/s12915-026-02707-3 (PMC13760885; doi:10.1186/s12915-026-02707-3)
Supplement: Supplementary file 2 [file 12915_2026_2707_MOESM2_ESM.docx]

**Fig. S3** SPDS chambers used in the experiment. The Soil Plant Daylit System (SPDS) chambers used in this experiment were designed to provide precise environmental control under semi-natural light conditions. Each chamber was enclosed with polymethyl methacrylate (PMMA) panels, allowing seasonal changes in light intensity and spectral quality to be represented more realistically than in conventional artificially illuminated chambers. Atmospheric CO₂ concentration was maintained using an automated CO₂ enrichment system. Pure CO₂ was supplied to each chamber to achieve the target concentration assigned to each SSP scenario. Deviations from the set point were automatically corrected through a feedback control loop connected to a GMW86P CO₂ transmitter. Air temperature was regulated by the integrated environmental control system. In the multi-chamber configuration used in this experiment, temperature was controlled using a centralized cooling system with auxiliary coils installed for individual chambers. The cooling package was housed in a low-profile weatherproof enclosure near the chambers. Environmental conditions were managed by networked microprocessor-based controllers, enabling centralized control and data acquisition. Air temperature inside each chamber was continuously monitored to ensure stable maintenance of the preset temperature assigned to each SSP scenario. For detailed information on the chambers, please refer to the link provided in parentheses (https://www.egc.com/day-lit-chambers/).
