# Supplementary material for: Drought resistance and post-rewatering recovery of Dendropanax trifidus under elevated CO2 and temperature are mediated by photoprotective regulation and carbon reallocation
Source: BMC Biol. 2026 Aug 14;24:237. doi: 10.1186/s12915-026-02707-3 (PMC13760885; doi:10.1186/s12915-026-02707-3)
Supplement: Supplementary file 3 [file 12915_2026_2707_MOESM3_ESM.docx]

| **Table S1** Scenario-specific environmental control parameters applied in nine SPDS chambers | | | | | | |
| --- | --- | --- | --- | --- | --- | --- |
| Month | Time  (o’clock) | SSP1-2.6 | | SSP3-7.0 | | SSP5-8.5 |
| May  Temperature  (℃) | 0~6 | 16.1 | 18.9 | | 19.8 | |
|  | 6~9 | 20.1 | 22.9 | | 23.8 | |
|  | 9~12 | 24.6 | 27.4 | | 28.3 | |
|  | 12~15 | 27.6 | 30.4 | | 31.3 | |
|  | 15~18 | 24.6 | 27.4 | | 28.3 | |
|  | 18~24 | 20.1 | 22.9 | | 23.8 | |
| Relative Humidity (%) | | 60 | | | | |
| June  Temperature  (℃) | 0~6 | 21.1 | 23.9 | | 24.8 | |
|  | 6~9 | 24.6 | 27.4 | | 28.3 | |
|  | 9~12 | 27.6 | 30.4 | | 31.3 | |
|  | 12~15 | 30.6 | 33.4 | | 34.3 | |
|  | 15~18 | 27.6 | 30.4 | | 31.3 | |
|  | 18~24 | 24.6 | 27.4 | | 28.3 | |
| Relative Humidity (%) | | 70 | | | | |

The experiment was conducted at the Climate Change Education Center of the Korea National University of Agriculture and Fisheries in Jeonju, South Korea. The Soil Plant Daylit System (SPDS) was used to simulate specific climate conditions.

Projections of CO_2_ concentrations were derived from the reduced-complexity climate–carbon-cycle model MAGICC 7.0 [59], while surface temperature projections were obtained from dynamically downscaled global UKESM1 projections for East Asia by 2080–2100 [37]. SSP1–2.6 was simulated with a CO_2_ concentration of 445 ppm and a temperature increase of 2.6 °C relative to the mean temperature in Jeonju, South Korea, from May to June. SSP3–7.0 was simulated with a CO_2_ concentration of 872 ppm and a temperature increase of 5.4°C. SSP5–8.5 was simulated with a CO_2_ concentration of 1,142 ppm and a temperature increase of 6.3°C.
