# Supplementary material for: Drought resistance and post-rewatering recovery of Dendropanax trifidus under elevated CO2 and temperature are mediated by photoprotective regulation and carbon reallocation
Source: BMC Biol. 2026 Aug 14;24:237. doi: 10.1186/s12915-026-02707-3 (PMC13760885; doi:10.1186/s12915-026-02707-3)
Supplement: Supplementary file 4 [file 12915_2026_2707_MOESM4_ESM.docx]

| **Table S2** Initial growth parameters of *Dendropanax trifidus* seedlings before SSP scenario and watering treatments | | | | |
| --- | --- | --- | --- | --- |
| SSP scenarios | Height (cm) | | RCD (mm) | |
| 1-2.6 | 38.0 | (0.89) | 5.34 | (0.15) |
| 3-7.0 | 36.6 | (0.62) | 5.15 | (0.11) |
| 5-8.5 | 38.0 | (0.78) | 5.22 | (0.18) |

Values enclosed in parentheses represent the standard error of the mean (n=18).
