# Supplementary material for: Drought resistance and post-rewatering recovery of Dendropanax trifidus under elevated CO2 and temperature are mediated by photoprotective regulation and carbon reallocation
Source: BMC Biol. 2026 Aug 14;24:237. doi: 10.1186/s12915-026-02707-3 (PMC13760885; doi:10.1186/s12915-026-02707-3)
Supplement: Supplementary file 5 [file 12915_2026_2707_MOESM5_ESM.docx]

**Table S3** Summary of statistical model specifications for all response variables

| **Response variable** | **Test type** | **Fixed factors** | **Repeated structure** | **Experimental unit and data handling** | **Error term** | **Post hoc method** | **Assumption checks** |
| --- | --- | --- | --- | --- | --- | --- | --- |
| F_v_/F_m_, PI_abs_ | Two-way repeated-measures ANOVA | SSP scenario, measurement stage, and SSP scenario × measurement stage | Repeated factor: measurement stage with five levels: early pre-drought stage (DS+14), late pre-drought stage (DS+20), mid-drought stage (DS+26), peak-drought stage (DS+29), and post-rewatering recovery stage (DS+43) | Chamber-level mean. Seedlings measured within each chamber were treated as subsamples and averaged before analysis.  n = 3 chambers per SSP scenario. | Residual error from chamber-level repeated observations | Bonferroni-adjusted multiple comparisons. | Residual normality, homogeneity of variance, and sphericity were assessed using Shapiro–Wilk, Levene’s, and Mauchly’s tests, respectively. |
| ABS/RC, DI_o_/RC, TR_o_/RC, ET_o_/RC, RE_o_/RC | Two-way repeated-measures ANOVA | SSP scenario, measurement stage, and SSP scenario × measurement stage | Repeated factor: measurement stage with five levels: DS+14, DS+20, DS+26, DS+29, and DS+43 | Chamber-level mean. Seedlings measured within each chamber were treated as subsamples and averaged before analysis.  n = 3 chambers per SSP scenario. | Residual error from chamber-level repeated observations | Bonferroni-adjusted multiple comparisons. | Residual normality, homogeneity of variance, and sphericity were assessed using Shapiro–Wilk, Levene’s, and Mauchly’s tests, respectively. |
| P_n,_ g_s,_ WUE_i_ | Two-way repeated-measures ANOVA | SSP scenario, watering treatment stage, and SSP scenario × watering treatment stage | Repeated factor: watering treatment stage with three levels: pre-drought stage, drought exposure stage, and post-rewatering recovery stage | One seedling per chamber was repeatedly measured. The chamber-level observation was used as the experimental unit. n = 3 chambers per SSP scenario. | Residual error from chamber-level repeated observations | Bonferroni-adjusted multiple comparisons. | Residual normality, homogeneity of variance, and sphericity were assessed using Shapiro–Wilk, Levene’s, and Mauchly’s tests, respectively. |
| RRG, RHG | Two-way repeated-measures ANOVA | SSP scenario, growth interval, and SSP scenario × growth interval | Repeated factor: growth interval with two levels: drought exposure stage and post-rewatering recovery stage | Chamber-level mean. Seedlings measured within each chamber were treated as subsamples and averaged before analysis. n = 3 chambers per SSP scenario. | Residual error from chamber-level repeated observations | Bonferroni-adjusted multiple comparisons. | Residual normality and homogeneity of variance were assessed using Shapiro–Wilk and Levene’s tests, respectively. Sphericity was not applicable because the repeated factor had only two levels. |
| Total Biomass, R/S ratio | Standard two-way ANOVA | SSP scenario, watering treatment stage, and SSP scenario × watering treatment stage | Not applicable. Destructive measurements were obtained from different seedlings harvested at each watering treatment stage. | Chamber-level mean of seedlings harvested independently within each chamber at each watering treatment stage. n = 3 chamber means per SSP scenario × watering treatment stage. | Residual error from chamber-level independent observations | Bonferroni-adjusted multiple comparisons. | Residual normality and homogeneity of variance were assessed using Shapiro–Wilk and Levene’s tests, respectively. |
| RWC | Standard two-way ANOVA | SSP scenario, watering treatment stage, and SSP scenario × watering treatment stage | Not applicable. Destructive measurements were obtained from different seedlings harvested at each watering treatment stage. | Chamber-level mean of seedlings harvested independently within each chamber at each watering treatment stage. n = 3 chamber means per SSP scenario × watering treatment stage. | Residual error from chamber-level independent observations | Bonferroni-adjusted multiple comparisons. | Residual normality and homogeneity of variance were assessed using Shapiro–Wilk and Levene’s tests, respectively. |
| SLA, XD | Standard two-way ANOVA | SSP scenario, watering treatment stage, and SSP scenario × watering treatment stage | Not applicable. Destructive measurements were obtained from different seedlings harvested at each watering treatment stage. | Chamber-level mean of seedlings harvested independently within each chamber at each watering treatment stage. n = 3 chamber means per SSP scenario × watering treatment stage. | Residual error from chamber-level independent observations | Bonferroni-adjusted multiple comparisons. | Residual normality and homogeneity of variance were assessed using Shapiro–Wilk and Levene’s tests, respectively. |
| TSS | Standard two-way ANOVA | SSP scenario, watering treatment stage, and SSP scenario × watering treatment stage | Not applicable. Destructive measurements were obtained from different seedlings harvested at each watering treatment stage. | Chamber-level mean of seedlings harvested independently within each chamber at each watering treatment stage. n = 3 chamber means per SSP scenario × watering treatment stage. | Residual error from chamber-level independent observations | Bonferroni-adjusted multiple comparisons. | Residual normality and homogeneity of variance were assessed using Shapiro–Wilk and Levene’s tests, respectively. |
| δ¹³C in leaves, stems, and fine roots | No inferential statistical test; descriptive and exploratory comparison only | Not applicable | Not applicable | One seedling per SSP scenario was analyzed during the post-rewatering recovery stage. n = 1 per SSP scenario. | Not applicable | Not applicable | Not applicable because inferential statistics were not performed due to n = 1 per SSP scenario. |

Notes:

a. SSP scenario was applied at the chamber level. Therefore, chamber-level observations were used as the experimental units for all inferential analyses. Individual seedlings or leaves measured within a chamber were treated as subsamples and were not used as independent experimental replicates.

b. Chlorophyll fluorescence parameters (F_v_/F_m_, PI_abs_, ABS/RC, DI_o_/RC, TR_o_/RC, ET_o_/RC, RE_o_/RC) P_n_, g_s_, WUE_i_, RRG, and RHG were analyzed using repeated-measures ANOVA because the same chamber-level experimental units were followed across measurement stages, watering treatment stages, or growth intervals.

c. Total biomass, R/S ratio, RWC, SLA, XD, and TSS were destructive measurements. Different seedlings were harvested at each watering treatment stage; therefore, these variables were analyzed using standard two-way ANOVA, not repeated-measures ANOVA.

d. Abbreviations: RRG, relative root collar diameter growth rate; RHG, relative height growth rate; F_v_/F_m_, maximum quantum yield of Photosystem II; PI_abs_, performance index on absorption basis; ABS/RC, absorption flux per reaction center; DI_o_/RC, dissipated energy flux per reaction center; TR_o_/RC, trapped energy flux per reaction center; ET_o_/RC, electron transport flux per reaction center; RE_o_/RC, electron flux to PSI acceptors per reaction center; P_n_, net photosynthetic rate; g_s_, stomatal conductance; WUE_i_, intrinsic water use efficiency; RWC, relative water content; SLA, specific leaf area; XD, xylem vessel diameter; TSS, total soluble sugar; R/S ratio, root-to-shoot ratio; DS, day of study.
